# WalkIm: Compact image-based encoding for high-performance classification of biological sequences using simple tuning-free CNNs

**DOI:** 10.1371/journal.pone.0267106

**Published:** 2022-04-15

**Authors:** Saeedeh Akbari Rokn Abadi, Amirhossein Mohammadi, Somayyeh Koohi

**Affiliations:** Department of Computer Engineering, Sharif University of Technology, Tehran, Iran; Taipei Medical University, TAIWAN

## Abstract

The classification of biological sequences is an open issue for a variety of data sets, such as viral and metagenomics sequences. Therefore, many studies utilize neural network tools, as the well-known methods in this field, and focus on designing customized network structures. However, a few works focus on more effective factors, such as input encoding method or implementation technology, to address accuracy and efficiency issues in this area. Therefore, in this work, we propose an image-based encoding method, called as WalkIm, whose adoption, even in a simple neural network, provides competitive accuracy and superior efficiency, compared to the existing classification methods (e.g. VGDC, CASTOR, and DLM-CNN) for a variety of biological sequences. Using WalkIm for classifying various data sets (i.e. viruses whole-genome data, metagenomics read data, and metabarcoding data), it achieves the same performance as the existing methods, with no enforcement of parameter initialization or network architecture adjustment for each data set. It is worth noting that even in the case of classifying high-mutant data sets, such as Coronaviruses, it achieves almost 100% accuracy for classifying its various types. In addition, WalkIm achieves high-speed convergence during network training, as well as reduction of network complexity. Therefore WalkIm method enables us to execute the classifying neural networks on a normal desktop system in a short time interval. Moreover, we addressed the compatibility of WalkIm encoding method with free-space optical processing technology. Taking advantages of optical implementation of convolutional layers, we illustrated that the training time can be reduced by up to 500 time. In addition to all aforementioned advantages, this encoding method preserves the structure of generated images in various modes of sequence transformation, such as reverse complement, complement, and reverse modes.

## Introduction

Classification, assigning input data to known classes of samples with similar features, has risen as an essential problem in biology studies so far. As a popular application of classification in biology, it categorizes creatures into various classes with specific evolutionary levels [1]. Depending on the type of creatures, the classification can be adopted in various fields; from evolutionary studies [2] to virus subtyping [3, 4], which is utilized for virology, epidemiology, and studying rate of disease progress and susceptibility drug treatment [5]. Subtypes (also known as clades or genotypes) are a crucial unit of viral nomenclature within a certain species, with each subtype representing a cluster of genetic similarities among isolates from the global population. Despite the fact that the genome sequences of the recognized subtypes of viruses can diverge, there is no obvious boundary between viral species. Several viral subtypes are clinically significant due to their ties to the variances in etiology, disease progression rates, and susceptibility to pharmaceutical treatments and vaccines. Since disease progression rates vary substantially among subtypes, newly discovered infections should be identified based on their genetic similarity to the curated reference subtypes [4]. Consequently, many studies try to propose automated methods for detecting viral subtypes using genomic data [3–5]. Moreover, classification, as a means of taxonomy analysis using meta-genomic reads, is applicable to biotechnology, ecology, bioremediation, and the medical field like diseases diagnosis and gut microbe studies [6]. Moreover, it can solve practical challenges in various areas, such as biofuels, biotechnology, food safety, agriculture, medications, and etc. [7]. Metagenomes data is obtained directly from the environment. As a result, the sequences of this dataset are not isolated and contain a variety of noises. Furthermore, since these datasets are made up of genomic reads, they necessitate use of sequencing procedures and alignment steps to specify their subtype. However, metagenomic analysis avoids these steps, as well as the use of cell cultures to characterize bacterial community composition derived from a specific environment. The later property permits bacteria to be cultured and isolated without the need for laboratory conditions, as many of them are difficult to culture.

According to the basic idea of classification approaches, they are categorized into two general classes [7, 8]; a) alignment-based methods, and b) alignment-free methods. As the alignment-based classifiers, BLAST [9], USEARCH [10], and REGA [11, 12] are considered as high-accurate classification methods [5], while their computational complexity, high run time, and resource consumption motivate [8] us to utilize alignment-free methods in most cases. Moreover, alignment-based methods necessitate some initial assumptions [8]. These assumptions (e.g. pre-defined substitution matrix or choosing a reference sequence for those methods based on phylogenetic likelihood [5, 13]) can highly impacts the classification result. As an another drawback of alignment-based classification methods, they need to compare any new input sequence with all previously classified ones, while this requirement limits the applicability of the methods for big data sets. Moreover, alignment-based classifiers suffer from high false-positive rate in the case of input samples with high mutation rates within each class. The later scenario usually happens for classifying sequences with limited similar fragments among samples [13].

On the other side, alignment-free classification methods can be categorized as either computational (deterministic) methods or learning-based methods. Regardless of the decision making approach (i.e. deterministic or learning-based), alignment-free methods are partitioned into two classes; feature-based and model-based methods [5, 13]. In feature-based methods, sequences are converted to feature vectors to feed the classification method. To perform this conversion, we can adopt either learning-based methods, such as like SVM and AdaBoost [8], or less computational process, such as multivector [14] and FCGR [15]. However, vector production from input sequences requires time-consuming pre-processing steps, and limits applicability of the classification method. Moreover, most vector-based methods, such as multivector [14] and RFLP-based (Restriction Fragment Length Polymorphism) [3], adopt heuristic approaches taking advantages of biological features of the input data. In this manner, despite their feature extraction capability, some of vector-based methods suffer from information loss. For example, k-mer frequency-based methods (e.g. FCGR-based method [15] and MLDSP [8]) eliminate spatial information, while multivector based methods ignore information of local patterns within the sequences (e.g. multivector [14]). As the last but not the least drawbacks of the k-mer based methods, these methods, such as MLDSP [8] and FCGR-based method [15], requires specific assumption on the size of K-mers and their distributions. All aforementioned issues raised for vector-based methods restrict their applicability. For instance, k-mer frequency-based methods, such as [15], or multivector methods, such as [14], cannot address problems involving motif finding, local information discovery within the sequences, locating transcription factor binding sites, or structural variations discovery, specially at the presence of high mutation rate within the input sequences. On the other hand, most aforementioned issues discussed for feature-based methods are raised for model-based methods as well. For instance, COMET [3], as a model-based classification method for HIV-1 subtypes, utilizes variable-order Markov model for all reference sequences and obtains likelihood of queries’ occurrence within each reference sequence. This method, similar to other methods in this category, necessitates selection of the best model order, adjustment of the window size, as well as the threshold value for recombination detection [5]. Although this method achieves good accuracy in classifying special taxa (HIV-1 subtypes), it is incompatible with other taxa (such as Influenza A) [3].

All the aforementioned challenges with various categorization approaches necessitate the development of genome classifier tools that are accurate, quick, and simple to use. In this regard, employment of Convolutional Neural Network (CNN), as a class of deep neural networks, has been proposed to address various concerns about accuracy [16]. Indeed, mostly fed by visual inputs, CNN is very successful in extracting essential features from the input images at the presence of noise [6]. During the last decades, the popularity of CNNs has been arising as a result of increased availability of computational resources, data sets, algorithms for training, and developed simple libraries for implementation [17]. On the other hand, since CNNs are generally successful in image processing, bioinformatics scientists came up with the idea of visualizing biological sequences as images [18–20]. Specifically, sequences visualization methods can encode various features of the input sequence, produce fixed size output image regardless of the input sequence length, and generate distinct signature from each bio sequence [18, 21]. However, these achievements come at the cost of reduced system performance [16]. This has led to the widespread adoption of vector formats on CNN, such as one-hot encoding, rather than image formats [5, 22]. However, the aforementioned advantages mentioned for visualizing biological sequences, as 2D or 3D images, are preserved. On the other hand, it should be noted that sequences visualization method can be also adopted in computational methods for encoding input sequences [15, 23].

So far, we have discussed various benefits of adopting CNNs feed by input images for genome classification, but it should be noted that some serious drawbacks, especially in the case of large input data, limit their applicability [17]. Performing convolution operations at the consequent layers causes high run time, power, and storage consumption, and so, have other side effects, such as environmental impact [24, 25]. Alongside, considering exponential growth of biological data sets as a result of improved sequencing technology, these issues become more critical. Although the processing time of training an ML model is not often considered within the run time of the classifier, its reduction has been targeted by many studied in the recent decade [5, 6]. To resolve the computational complexity of CNN architecture, in this paper, we propose to migrate the implementation technology from electrical to optical domain to considerably improve run time and energy consumption of CNNs. Theoretically, we can achieve the computation speed of light and save energy up to 90%, and thus, reduce environmental degradation [26]. This solution is possible due to the easy implementation of convolution with two simple lenses in the field of optics and the use of data as an image [26], and a lot of works have been done to implement it in the both form of free space [27–29] and on-chip [25, 26]. In this way, by converting sequences to standard images format, this solution can be used, and in addition to the very good extraction of features that CNN provides, the best time and the best energy consumption can be achieved compared to all other methods of ML.

All aforementioned advantages for sequences visualization motivate researchers to visualize bio sequences in the form of 2D or 3D images [15, 30–32]. Theoretically, all kinds of K-mer histogram of sequences [6, 8, 30], one hot representation, representation methods based on physicochemical properties [33], representation methods based on a combination vector of several types of information [34], and DNA-walk representation can be adopted to encode biological sequences as 2D images. Of course, it should be noted that methods like [33] that emphasize physicochemical properties are often developed for protein sequences, since the physicochemical properties of amino acids are much more diverse, and therefore, create a rich set of information. However, it should be mentioned that the corresponding generated images do not necessarily satisfy the standard image format required for their adoption in CNNs, which is a 2D arrays of color triplets whose values are in the range of 0 and 255. In addition, there are some flaws in genomic visualization techniques, as reported in Table 1, for some well-known visualization methods. As mentioned in this table, in [6], a one-dimensional matrix containing the K-mer frequencies feeds two types of CNN and DBN networks, similar to the encoding scheme adopted in [8]. This matrix contains 4^K^ entries of decimal numbers, each represents the frequency of the corresponding K-mer’s state. Although the proposed K-mer counting method [6] can create a vector in particular and limited dimensions, its classification accuracy is significantly affected by the length of the substrings, or the value of k. As a result, reducing vector dimensions is limited to guarantee adequate precision. As another challenging issue for this encoding, it should be mentioned that for the small values of k, as usual for most biological tools, the vector’s entries are extremely diverse. Therefore, information loss occurs as the result of mapping the vector’s entries to the limited range of [0, 255] assumed for common image formats. Finally, since this encoding scheme loses the spatial information of the sequences, it cannot be adopted in a variety of applications, such as motif finding.

**Table 1:** Comparison of well-known sequence encoding methods; S.I (Standard Image format).

Encoding methods	Size	Local inf.	S.I. compatibility	More
K-mer (FCGR) [6, 8]	4^k^	No	No–Values > 255	The best size of k is not clear—Does not store local information
Spatial_K-mer (FCGR) [30]	(L×4^k^)/CS	Yes	No–Values > 255 –Image Dim	The best size of k and chunk size (CS) is not clear
DNA-Walk_xy-plane_ [31, 32, 35]	≤L^2^/4	Yes	No–Image Dim.	data lost (because path overlap)
DNA-Walk_quadrants_ [31, 36]	≤ L^2^/4	Yes	No–Image Dim. Pix locate	-
One-hot [22, 31]	4L	Yes	No–Image Dim.	encoding without any processed information
Integer [5, 31]	L	Yes	No–Image Dim.	encoding without any processed information

To overcome the aforementioned issues, various methods were proposed, such as Spatial_K-mer [30], to encode locations as well as frequencies of the K-mers. For this purpose, the sequences are first split into chunks of constant length, and then, the K-mer frequencies for each substring are determined to preserve the local information of the string. However, extracting K-mer frequencies for each substring increases the vector size, compared to the traditional K-mer counting method, while the vector’s length is not fixed and is determined by the input sequence length. Furthermore, similar to usual K-mer frequency encoding methods, the vector’s entries vary in a wide range, which is not the case for the common image formats.

To preserve spatial information of the sequence, DNA-walk [32–34] has been proposed, which produces an image containing an observable and recognizable pattern. In general, in this method, each nucleotide is represented as a two-dimensional vector, although it can be extended to three-dimensional versions or higher. To encode the sequences, each letter of the sequence is substituted by the relevant vector, and so, consequent vectors form a path for the whole sequence. Although DNA-walk produces meaningful image which can be even recognized by humans, it suffers from various drawbacks, as follows: a) the image size increases by the length of the sequence, b) data loss occurs as the result of subsequent vectors overlap, depending on the directions of the corresponding nucleotides’ vectors. Specifically, overlap scenarios happen when subsequent vectors are perpendicular to each other, as happens for the method presented in [32], although there would be no data loss if the vectors do not return, as proposed in [34]. On the other hand, by quantizing vectors’ length in the images, the DNA-Walk presented in is unable to distinguish various vectors with angle difference less than 45 degrees.

Finally, considering sequence classification applications, several numerical encoding approaches, such as substituting various nucleotides with integer values [5] or One Hot encoding [22], have been proposed. However, since the size of the resultant encoded data depends on the sequence lengths, these methods cannot produce proper images for an optical CNN architecture. As reported in Table 1, the column "Size" in this table indicates the vector size for each method depending on string length or K value (for k-mer based methods). The "Local inf." column also shows whether the method keeps local data. The "S.I compatibility" column shows whether the encoding method is compatible with the Standard Image (S.I) format (3D matrix for RGB channel with finite integers in the range of 0 to 255).

To resolve most concerns mentioned in Table 1, this paper presents a novel image generation method to preserve key aspects of biological sequences. Designing an encoding approach based on DNA-walk, we can restrict output image sizes despite rising input sequence lengths and, most crucially, eliminate usage of prior knowledge or pre-processing of input sequences.

Our goal, based on what has been discussed, is to develop an encoding method that includes all of the following features:

- Developing an image-based encoding strategy that takes advantage of CNNs’ perfect image-processing abilities.
- Images are generated in typical image formats
- Images include the sequence’s general and local information
- Image size that grows slowly in proportion to the sequence length.

More features are discussed in the following sections. The next step is to employ our new images in a very basic CNN with the goal of accurately classifying many different types of data sets in a generalized manner without any special settings for each data set. In reality, additional accuracy can be attained by fine-tuning CNN for each data set. In this study, we focus on DNA, but it can be generalized to RNA and Protein too.

## Materials and methods

**Fig 1:**
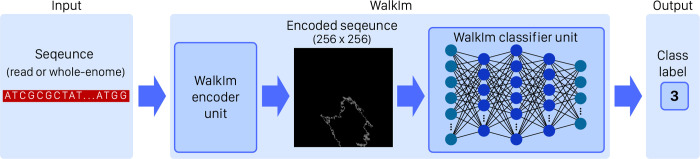
Schematic of WalkIm method; WalkIm consists of two units; encoder unit and CNN-based classifier unit.

Designing deep learning methods involves two parts: a) input data pattern and its encoding, and b) neural network design, while optimized co-design of input coding method and neural network affects output accuracy. Although most studies focused on optimized network design to achieve high accuracy, it comes at the cost of increased network complexity, as well as high runtimes of learning and evaluation processes [6, 8]. As discussed in this paper, the type of input data and its encoding approach can help us alleviate these problems. However, it should be emphasized that the encoding method should not be overly complicated, which in turn increases pre-processing time. So in this paper, a novel encoder-classifier method, named as WalkIm (DNA-walk based Image), is introduced to resolve the aforementioned issues. As shown in Fig 1, WalkIm consists of an encoding unit to perform input data processing, and a CNN unit to accomplish data classification. As follows, we explore input data encoding and CNN design of WalkIm in more details.

### Input data

Since we do not customize WalkIm for a specific data type, to evaluate its accuracy, we prepare two assessments by addressing three frequently referred usages of sequence classification methods in various levels of evolution: a) viral classification [5], b) bacterial classification based on metagenomics data [6], and c) metabarcoding classification [37]. The viral classification itself includes five tests to classify various types of Dengue, Hepatitis B, Hepatitis C, HIV-1, Influenza A, and corona. Except corona, the corresponding data sets are collected as described in [5]. The corona data set is collected separately, with access details listed in the section “Data” of S1 File. It should be noted that the conditions for downloading data sets are given in [5], and as a result, the number of files available for each data set may vary based on the download time. Therefore, our data sets have different numbers of samples, compared to those of [5]. Specifically for the HIV (1) data set, with 37 categories mentioned in [5], we have imported 36 categories, while there was no sample for one of the categories mentioned in [5]. Bacteria taxonomy consists of four tests to classify the same samples generated using Amplicon (AMP), the next generation sequencing technology, into four levels of evolution (i.e. class, order, family, and genus), while these samples are accessible from [6]. The last data set is a barcoding data set consisting of cytochrome c oxidase subunit I (COI) DNA barcode sequences to taxonomic kingdoms [37]. Specifications of these data sets are summarized in Table 2, while all access information for these data sets are provided in section "Data" of S1 File.

**Table 2:** Summarized information of utilized data sets; * one class of this data set has over 300 thousand samples, and we take a random subsample of it with 2000 samples to balance the data set, as described in [37].

Data sets		#Classes	#Samples	Min. sequences’ length	Max. sequences’ length
Viral	Dengue	4	5446	10161	11195
	Hepatitis B (1)	8	6560	3182	3257
	Hepatitis B (2)	13	7443	3182	3257
	Hepatitis C	9	2017	24751	24751
	HIV-1 (1)	12	7669	19685	24307
	HIV-1 (2)	36	11391	19685	24307
	Influenza A (1)	56	121454	173	2867
	Influenza A (2)	113	123050	173	2867
	Corona	7	874	15572	30818
Metagenomics*		3	28000	210	257
		20			
		39			
		100			
Metabarcoding (COI DNA barcode)		5	9406	93	2070

### WalkIm encoder unit

**Fig 2:**
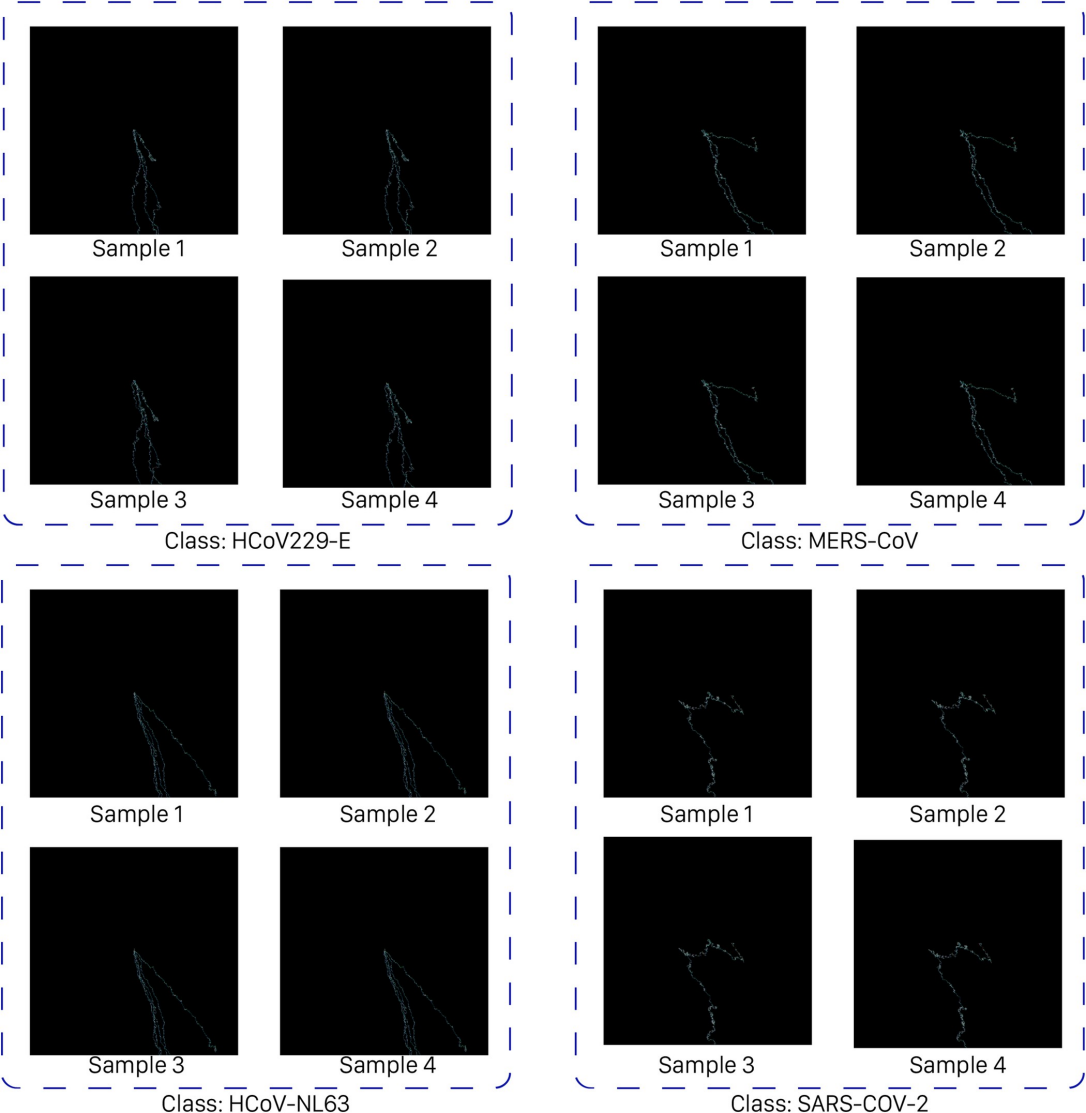
Recognizable similarities of several images generated by DNA-Walk for four sample genomes of the four groups of coronavirus.

As the encoding unit of WalkIm, we modify DNA-walk encoding method to eliminate special process, as well as pre-knowledge requirements of input data and its distribution. This feature enables WalkIm to target any type of input sequences, either biological or non-biological text-based data. Moreover, outputs of the WalkIm encoding unit represent signature of the input sequences, which are distinguishable for varying human beings. In this manner, taking advantages of input signature, it avoids complex CNN structure to perform classification. This property is illustrated in Fig 2, which represent several sample images from several corona categories.

As the key advantages of DNA-walk representation, we can mention that it preserves locational information, and depicts nucleotides distribution within various fragments of the sequence, as well as the whole sequence. However, it offers various variations as follows; the earlier model of DNA-walk uses four main directions (i.e. west, east, north, and south) for representing each nucleotide, and hence a DNA (or RNA) sequence is plotted by consequent unit vectors in these directions [32, 35, 38]. As its main drawback, overlapping and crossing of the curves, representing DNA segments, cause information loss. As a modified version of the DNA-walk representation, [36] proposes adoption of four vectors with direction angles in the range of 0 to 90 degree to avoid path return. Despite this improvement, all variations of DNA-walk encoding methods lead to output images whose size depends on the length and distribution of the nucleotides. Nonetheless, almost all variations of DNA-walk encoding methods achieve good accuracy in comparing and classifying biological sequences [32, 39].

**Fig 3:**
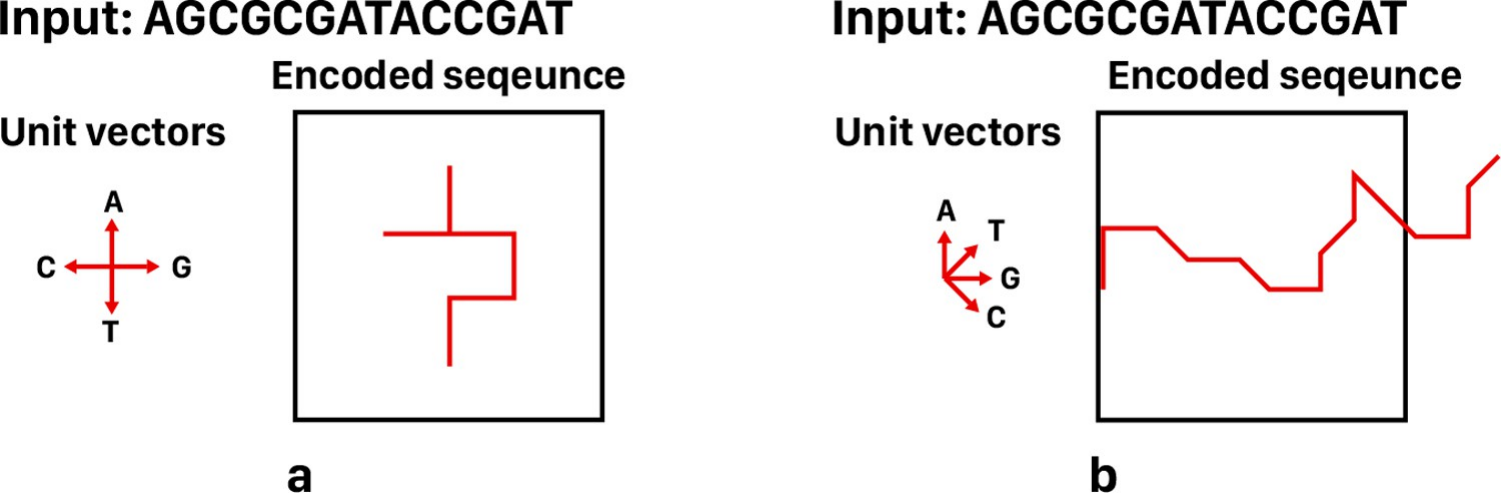
Some challenges of applying DNA-walk method; a) untraceable path due to various overlaps of DNA pathways, which results in data loss, b) infinite space required for curve generation, which makes it impossible to store the generated image within a confined space.

As discussed, various versions of DNA-walk encoding method are designed to address its main drawbacks, such as information loss. However, even its recent versions still face some challenging issues. Specifically, Fig 3 shows the growing trend of DNA-walk encoding methods and their main challenges. For example, various studies set target to avoid probable overlaps of DNA pathways, as the main drawback of DNA-walk representation, by preventing loop formation through the pathway [40, 41]. But this improvement comes at the cost of increased image size. On the other hand, various studies deal with DNA-walk encoding as a variable-size visual representation, since its output image’s size depends on the input sequence pattern. To address these challenges, and many other ones, WalkIm encoding method is proposed to generate fixed-size output images, with fixed range of pixel values, which can be fed to any image-based CNN classifier. Finally, although many studies target accuracy improvement of DNA-walk encoding, a few of them [32, 39] discuss variety of its applications.

WalkIm’s encoding unit is designed to generate standard output image format (i.e. discrete space, fixed size image with pixel values in the range of 0 to 255). It should be noted that it has two versions; 3 layers for RGB color channels and 1 layer for Grayscale format. Every pixel of the image, except for those on the sides, has 8 neighbor pixels alongside its sides and corners, as shown in Fig 3, and so has 8 directions to move forward. As follows, various steps of WalkIm’s encoding unit are presented:

1. Consider a square of size M×M.
2. Select the central pixel with coordinates O = (M/2 and M/2) as the starting point to facilitate initial move in any direction.
3. Assign each square corner to one of the four possible nucleotides (i.e. A, C, G, and T).
4. Pars the input DNA sequence (from 3’-end to 5’-end). By reading each nucleotide, move toward the corresponding direction by one pixel, and increase the pixel value by a constant value. This step is done for a grayscale format in a single-layer 2D matrix. While for a three-layer format, three of the four letters are assigned to each of three layers of RGB image, and the fourth letter is assigned to two of three layers.
5. If next move hits the image sides, return to point O and go to step 4. O.W., continue to step 6.
6. End of encoding.

**Fig 4:**
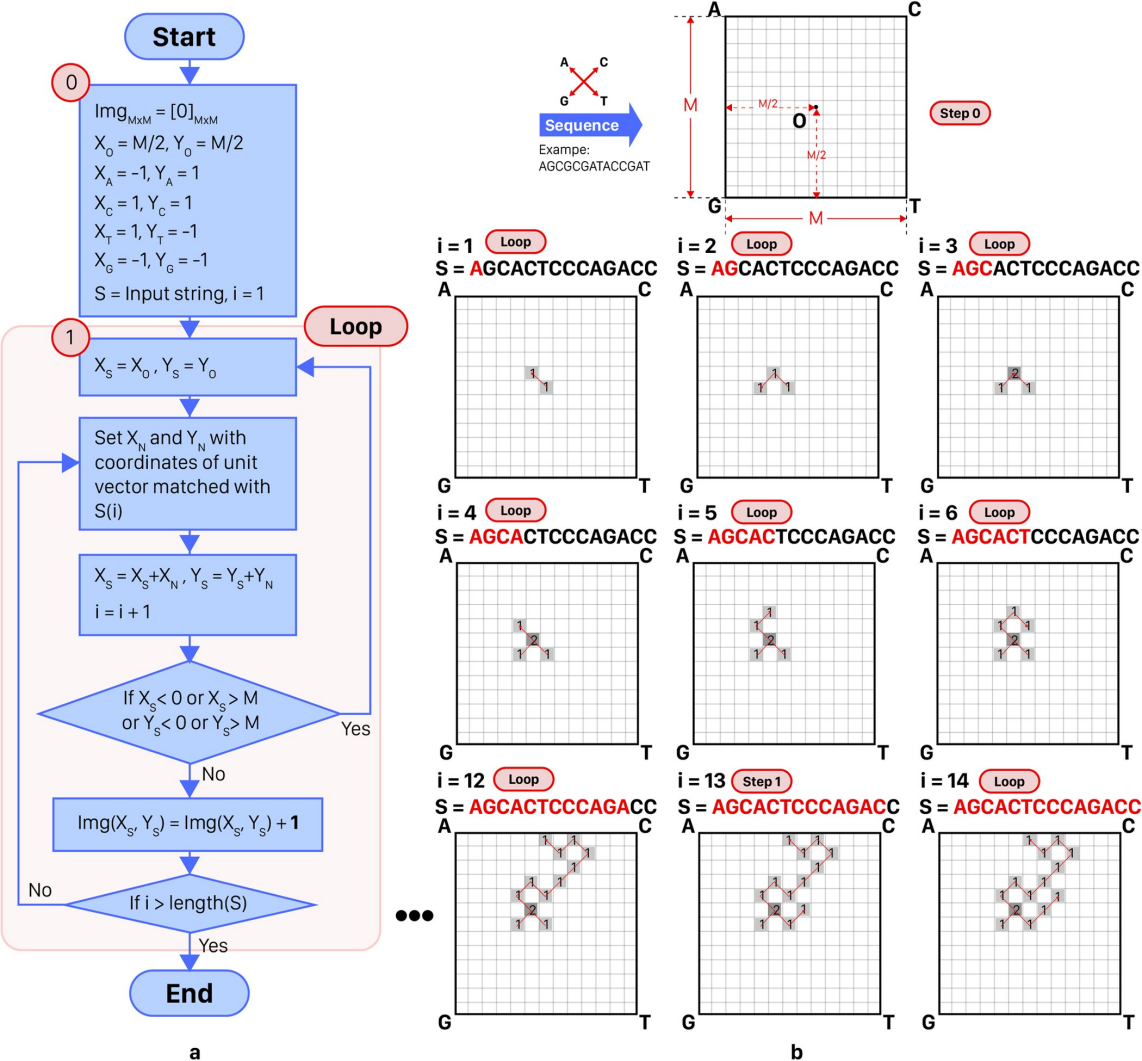
a) Flowchart of WalkIm encoder unit, b) Sample run of WalkIm encoder. This image is set to grayscale format without loss of comprehensiveness, and it is similar to its format. In addition, the constant value at each step is considered as one.

For more clarity, the above procedure is schematically depicted in Fig 4. It is worth noting that the constant value, added to the matrix cells in each step, can vary. For high image resolution and contrasts, it is recommended to set this value to more than 10 (instead of one). In this case study, this value is set to 255. Although the produced values in the matrix may exceed 255, the maximum pixel value, Python automatically converts these values to the range of 0 to 255 when saving the image content, and hence, it preserves the information during the encoding procedure.

**Fig 5:**
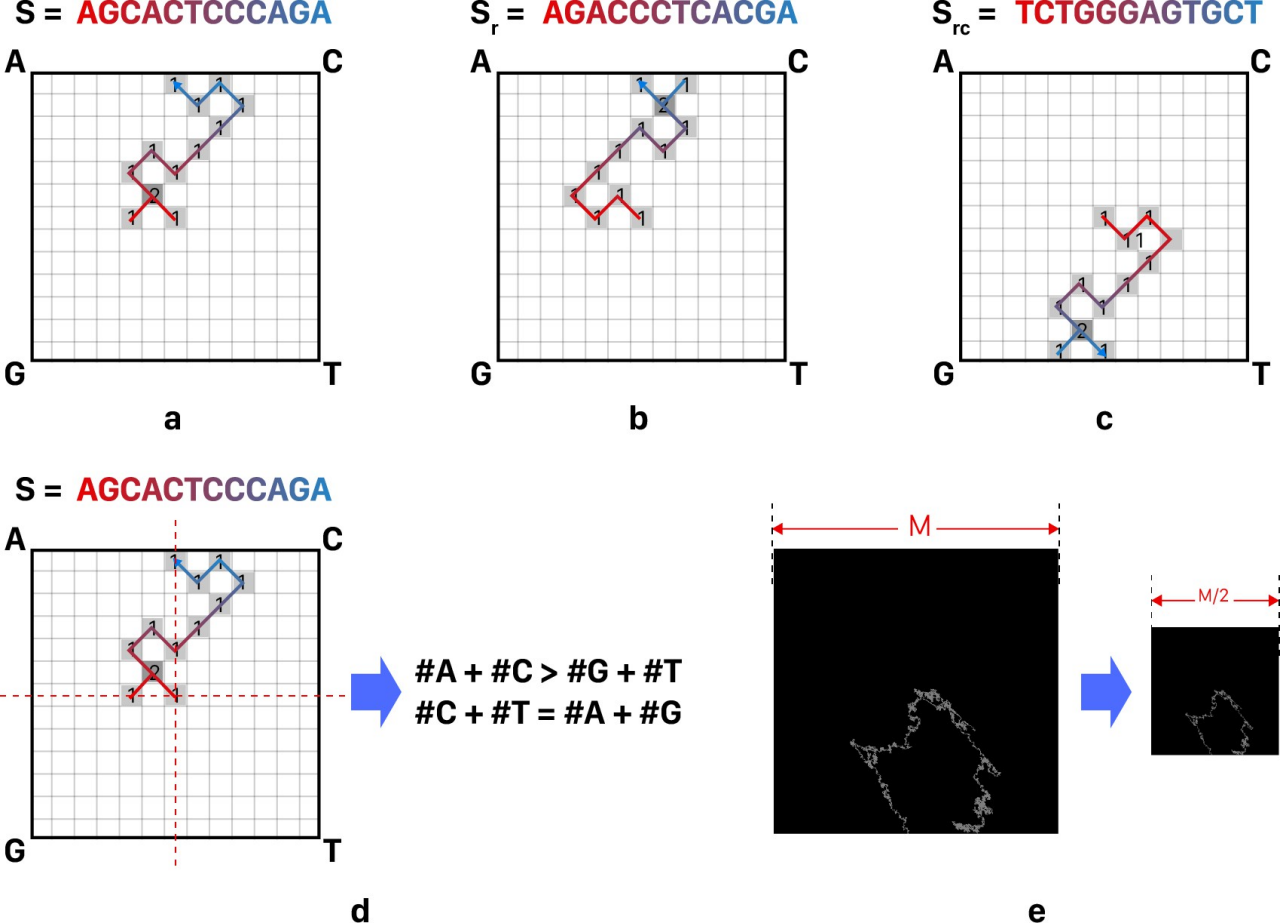
Some key features of WalkIm encoder; a) usually, input sequences can be rebuilt (i.e. decoded) from the encoded image and the generated shape is traceable, b) the shape generated from the reversed sequence (S_r_) is symmetric about the x axis, compared to its original shape, c) the shape generated from the reverse-complemented sequence (S_rc_) is symmetric about the x axis, compared to its original shape, d) the generated shape contains some statistical information.

Main points of the proposed encoding method are described as follows:

1. Considering non-binary pixel values, overlaps of the pathway can be mostly traced, and hence, information loss is avoided, unlike previous versions of DNA-walk [32, 35]. This property is illustrated in Fig 5A.
2. In the case that each pair of complement nucleotides (i.e. (C and G), (A and T)) is assigned to two corners on the same diameter, the encoded sequences, either from 3’-end to 5’-end or from 5’end to 3’-end, would be in similar shapes, as well as symmetric with respect to the central pixel of the square, as shown in Fig 5B. This also happens for the reverse complement sequence, as shown in Fig 5C.
3. Overall distribution of nucleotides throughout the input sequence and their statistical features can be visually analyzed by studying the pathway directions, as shown in Fig 5D. For example in this sample figure, the instance shape is placed on top half of the image which means the numbers of A and C are more than those of G and T, Moreover, there is an equal number of pixels in the left and right halves and a reciprocating path is created between the two halves, which means that both halves of the sequence have almost the same numbers of A and G, compared to those of C and T.
4. Encoding an input sequence within a square with specific size can be performed in two ways; a) the proposed encoding method generates coded image within a square with desired size, or b) it produces a larger image, and afterward, downscales it to an image with the desired size. In the latter case, while reducing the number of times the pathway hits the sides of the square, the overall shape of the pathway is preserved, as shown in Fig 5E. In this manner, we can increase classification accuracy with providing more directions for various moves.

### WalkIm classifier unit

Due to the nature of genomics data, it is not possible to adopt popular and powerful convolutional networks (e.g., LeNet [42], AlexNet [43], GoogLeNet [44], ResNet [45], or VGG [46]) straightforwardly [5]. This complexity arises from the different data dimensionality of genomes, compared to traditional image formats. Specifically, one-dimensional genomes cannot be easily feed to convolutional networks operating on two-dimensional images. On the other hand, although various studies propose restructured CNNs [5, 6, 17] to be compatible with genomic data, they reduce data processing capability of these networks.

In this manner, we propose a novel encoding method to facilitate adoption of powerful convolutional architectures for genomics data processing. Specifically, we encode each sequence as an image and feed it to a CNN. Moreover, instead of using complex convolutional architectures, we adopt a simple and shallow convolutional neural network, named as WalkIm classifier unit, for four reasons:

- Powerful encoding method: To emphasize the capability of WalkIm encoding method, and its impact on the classification accuracy, we take advantages of a few convolutional layers, unlike popular DNNs.
- Facilitating optical implementation: Taking advantages of optical implementation of convolutional layers [26, 27], the proposed neural network can be easily implemented in the optical domain. Specifically, Well-known 4f optical correlator is a common architecture for performing convolution operation in free space optics [27, 47]. This system is based on the notable Fourier transforming properties of converging lenses. Specifically, the structure of a 4f correlator system consists of an input plane, first lens, Fourier plane, second lens, and output plane [47]. Detailed explanations on this configuration are provided in S1 File section "Optical CNN setup."
- Facilitating PC-based implementation: Due to the large size encoded images and the massive data sets, a shallow convolutional neural network is proposed to enable network implementation on normal desktop computers.
- Eliminating parameter initialization: As a key advantage of WalkIm, it does not require initialization of network parameters for varying (or new) data sets.

**Fig 6:**
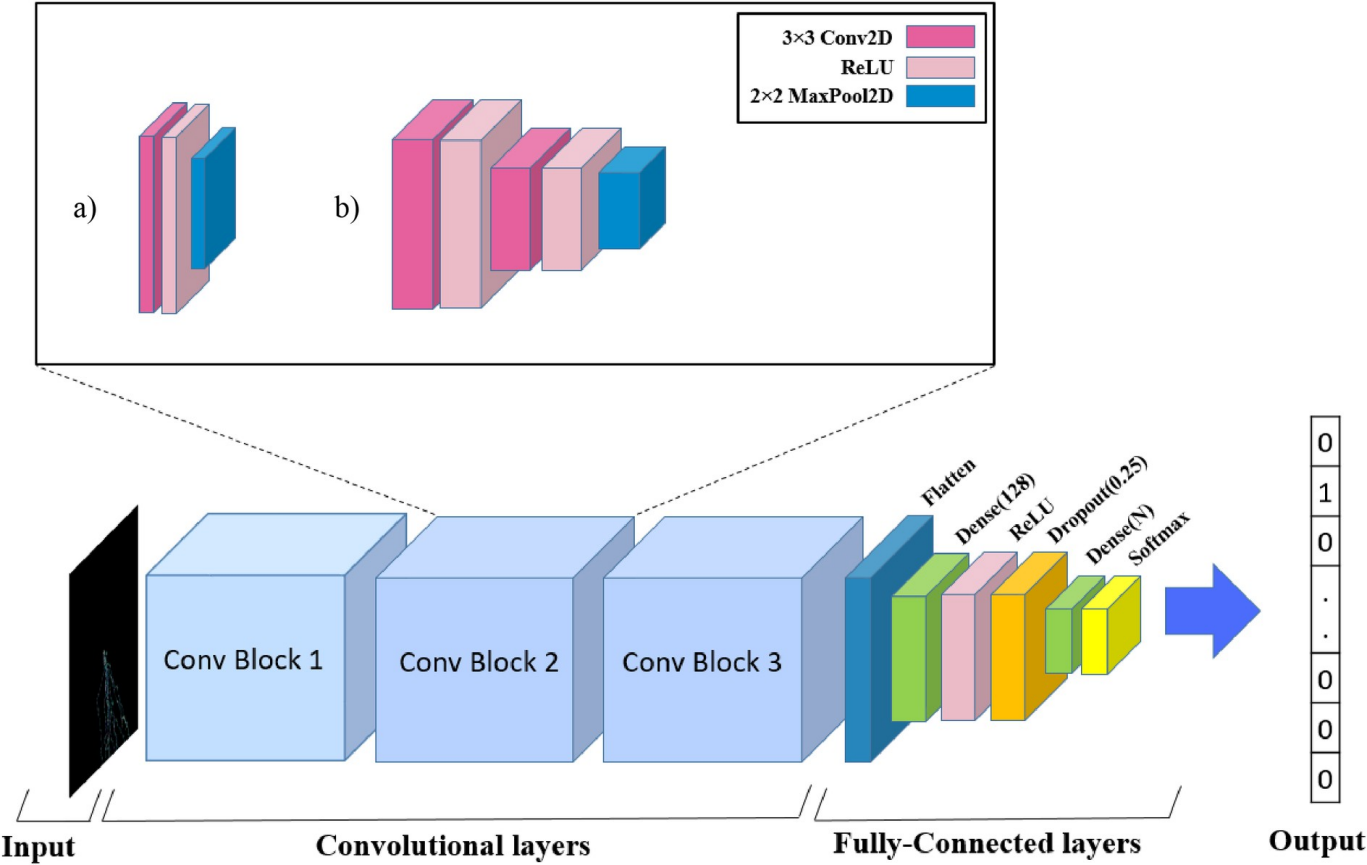
The proposed structures for WalkIm classifier unit;a) simple CNN structure named as CNNsimple, b) deeper CNN structure named as CNNcomplex.

Considering all aforementioned explanations, we provided the WalkIm classifier unit in two versions, simple and a little deeper, in order to investigate the effect of network depth on the classifier’s accuracy. It should be noted that almost all recently developed neural network for sequence classification [5, 6] are customized with specific parameters values for each data type and species to achieve acceptable classification accuracy. However, as a key advantage of our proposed encoding method, we do not impose such network settings. In this manner, to clarify the power of the WalkIm encoder, we just employed the most basic networks to attain similar accuracy, compared to the alternative tools. Of course, so much better results can be obtained by configuring a classifier unit for each category. The architectures of two versions of WalkIm networks proposed for genome classification is presented in Fig 6. These are convolutional classifier models whose input images are produced by WalkIm encoder. It is worth noting that unlike most existing methods, such as VGCD and [48] size of the input images in WalkIm network does not depend on genome length. Specifically, without loss of generality, we resize encoded images to 256 by 256. In this manner, our input images have dimensions of 256 × 256 × 3 (for RGB format) and 256 × 256 × 1 (for grayscale format), while these sizes can be increased or decreased, with no impact on the classification accuracy.

### CNN characterization

The proposed CNN architecture produces a vector P of size 1 × N, where N is the total number of classes (i.e., viral subtypes) for the given problem, and entry P_i_ ∈ P (1 ≤ i ≤ N) represents the probability of a given genome, encoded and fed as the input image to CNN, belonging to the i-th class.

As depicted in Fig 6, there only exist two parts dedicated to a) the convolutional layers, performing feature extraction, and b) the fully-connected layers, predicting the genome subtype, based on the features extracted by the convolutional part. The detailed explanations are provided as follows.

- Convolutional layers: Input images are fed to three consequent and similar convolutional blocks (shown as Conv Block1, Conv Block2, and Conv Block3), each has one and two 2D convolutional layers for simpler network (CNN_simple_ in Fig 6A) and more complex network (CNN_complex_ in Fig 6B), respectively. Each convolutional layer is followed by a ReLU (Rectified Linear Unit) activation layer to improve training performance [49].
  In the proposed CNN architecture, each convolutional layer convolves the corresponding input image with a set of learnable filters whose coefficients are learned through the network training process. In WalkIm network, filters of size 3 × 3 are assumed, while the number of filters is increased by a factor of two from each convolutional block to the next. Specifically, it varies from 8 (for CNN_simple_ in Fig 6A) and 64 (for CNN_complex_ in Fig 6B) for the first convolutional block to 32 and 256 for the third one.
  The rectified linear activation layers (ReLU), following the convolutional layers, introduce non-linearity to reduce over-fitting. Specifically, ReLU reduces the vanishing gradient problem, and avoids back propagation errors, while it is much faster, compared to sigmoid activation function.
  As the last layer of convolutional block, 2D pooling layers follow ReLUs and perform max-pooling operation with the pooling filter of size 2 × 2 and the stride of 2 for both simpler and complex networks. Although pooling operation reduces the input size, it extracts characteristic genomic features and propagates them to the dense layers. Finally, the output of the last max-pooling layer is converted into a 1D feature vector to be used by the classifier part of the network, as follows.
- Classifier layers: As the classifier part of the network, WalkIm takes advantages of two dense layers with the decreasing number of neurons, from 128 neurons in the first dense layer to N neurons in the second one, where N is the number of subtypes within each data set. The first dense layer, followed by a ReLU layer and a dropout layer, feeds the last layer implementing softmax activation function. Finally, WalkIm produces probabilities of a given genome sequence belonging to each class. It is crystal clear that the genome is classified as the subtype with the highest probability value.

### Training parameters

To evaluate classification accuracy and runtime of WalkIm network for various data sets, five-fold cross-validation for viral [5] and metabarcoding [37] data sets, and ten-fold cross-validation for metagenomics [6] data sets are performed. In each experiment, the network is trained for a maximum of 30 epochs, unless the early stopping condition is fulfilled, when the training is stopped after several successive epochs with no training improvement. According to our simulation studies, usually, the training converges at about 15 epochs. Adam optimizer [50] with the learning rate of 0.001 is adopted to minimize the categorical cross-entropy loss function, and the mean squared error is utilized to measure performance of the model. The batch size is assumed to be 64, except for the large number of training samples, where we assume the batch size of 256. Finally, it should be noted that the aforementioned values of hyper parameters are determined in a trial-and-error manner balancing training time versus training performance.

It is worth noting that as a key advantage of WalkIm classifier, values of CNN parameters, e.g. filter size w, do not depend on the length of input genomes and can be constant for all data sets. The size of CNN input vector (i.e. n) is equal to the product of image’s dimensions. While, the size of output vector (i.e. N), generated by the CNN, is equal to the number of virus subtypes to be predicted.

### System specification

The proposed CNN is implemented in Python 3.6, with the Keras library running on top of TensorFlow. The experiments are performed on a normal desktop computer (i7-6500 2.5 GHz CPU, 8 GB RAM) with GeForce GTX 920M GPU equipped with 2 GB of DDR3 RAM.

## Results and discussion

### Metrics for comparison

Performance of a classifier is generally measured by popular metrics combining four basic metrics, i.e. TP (True Positive), TN (True Negative), FP (False Positive), and FN (False Negative). TP is the number of cases correctly identified as members of a class. FP is the number of cases incorrectly identified as members of a class. TN is the number of cases correctly identified as non-members of a class. And finally, FN is the number of cases incorrectly identified as non-members of a class. We also use five reputable metrics, based on four basic ones [5]. Specifically, these five reputable metrics, i.e. sensitivity (Se), specificity (Sp), precision (PREC), accuracy (ACC), and F1-score (F1) are defined by Eqs 2 to 5 respectively.


$$Se=\frac{TP}{TP+FN}$$ Eq 1

$$Sp=\frac{TN}{TN+FP}$$ Eq 2

$$PREC=\frac{TP}{TP+FP}$$ Eq 3

$$ACC=\frac{TP+TN}{TP+TN+FP+FN}$$ Eq 4

$$F1=\frac{2TP}{2TP+FP+FN}$$ Eq 5

For a more detailed description, we should note that Se indicates the percentage of a class members correctly identified as members of that class. Sp shows the percentage of a class non-members correctly identified as non-members. PREC indicates the percentage of items identified for a class and actually belong to it. ACC indicates the percentage of correct diagnoses have been made in total (whether the class members or non-members are correctly identified), and finally, F1-score is the harmonic mean of precision and sensitivity values, and it is advisable when a balance between precision and sensitivity is required and many actual negatives exist. In addition to these metrics, as follows, we also present confusion matrices obtained from each test to analyze WalkIm performance and features of data sets in Section “Confusion matrices” of S1 File.

As a comprehensive simulation study, WalkIm method is investigated and analyzed from several aspects. Specifically, we have created some comparative simulation scenarios as follows:

1. First, we use CNN_simple_ to compare the grayscale and RGB modes of the created images in terms of classification accuracy. Afterwards, considering the more accurate image mode from the previous analysis, CNN_complex_ is employed to investigate accuracy of WalkIm encoding method for various data sets in more details. In this manner, the impact of image type and network depth on classification performance can be deeply analyzed. As the key advantage of the proposed encoding method, these CNNs are adopted, with no specific initial settings, to analyze performance of WalkIm encoding. They are fed by data sets of three types of biological data, i.e. virus classification data [5], metagenomics data [6], and metabarcoding data [37], with variations in the number of samples, categories, and string lengths.

2. We estimate the training runtime of the optical CNN architecture, adopting WalkIm method, for each data set and compare it to the values published in the reference papers.

### Performance comparison

As discussed above, we adopt three types of data from three most relative papers [5, 6, 37] to analyze encoding capability of WalkIm feeding the neural networks and for a fair comparison, similar comparison scenarios are adopted. We first evaluate both grayscale and RGB formats with CNN_simple_, as indicated in the previous section, while the comparison metrics are shown in Tables 3 to 5. At the next step, depending on which image format works with the simpleset network (i.e. CNN_simple_), we analyze it in more details with a deeper network (CNN_complex_). These results are also given in Tables 3 to 5 for each data set, while the corresponding discussion are presented as follows. Moreover, confusion matrices are reported in the appendix’s "Confusion matrices" section.

**Table 3:** Classification performance measures achieved by various virus classifiers.

Data set	Method	Se	Sp	PREC	ACC	F1
**Dengue**	WalkIm_grayscale-simple_	1.000	1.000	1.000	1.000	1.000
	WalkIm_RGB-simple_	0.999	1	0.999	1	0.999
	WalkIm_RGB-deep_	0.998	0.999	0.998	0.999	0.998
	VGDC	1.000	1.000	1.000	1.000	1.000
	CASTOR	1.000	1.000	1.000	1.000	1.000
**Hep. B (1)**	WalkIm_grayscale-simple_	0.996	0.999	0.996	0.999	0.996
	WalkIm_RGB-simple_	0.996	0.999	0.996	0.998	0.996
	WalkIm_RGB-deep_	0.995	0.998	0.995	0.998	0.995
	VGDC	0.999	1.000	0.999	0.999	0.999
	CASTOR	1.000	1.000	1.000	1.000	1.000
**Hep. B (2)**	WalkIm_grayscale-simple_	0.933	0.983	0.926	0.982	0.925
	WalkIm_RGB-simple_	0.933	0.983	0.926	0.982	0.925
	WalkIm_RGB-deep_	0.929	0.986	0.927	0.981	0.927
	VGDC	0.954	0.988	0.953	0.987	0.952
	CASTOR	0.949	0.985	0.945	0.986	0.945
**Hep. C**	WalkIm_grayscale-simple_	0.970	0.987	0.967	0.990	0.968
	WalkIm_RGB-simple_	0.972	0.988	0.969	0.991	0.97
	WalkIm_RGB-deep_	0.969	0.988	0.970	0.981	0.969
	VGDC	0.996	0.999	0.996	0.999	0.996
	CASTOR	0.996	1.000	0.996	0.999	0.996
	COMET	0.958	0.984	0.962	0.984	0.957
**HIV-1 (1)**	WalkIm_grayscale-simple_	0.941	0.965	0.933	0.98	0.935
	WalkIm_RGB-simple_	0.920	0.938	0.903	0.967	0.905
	WalkIm_RGB-deep_	0.925	0.985	0.946	0.974	0.934
	VGDC	0.979	0.993	0.978	0.995	0.978
	CASTOR	0.942	0.985	0.940	0.984	0.940
	COMET	0.904	0.964	0.862	0.975	0.870
**HIV-2 (2)**	WalkIm_grayscale-simple_	0.908	0.995	0.921	0.986	0.91
	WalkIm_RGB-simple_	0.904	0.962	0.904	0.975	0.892
	WalkIm_RGB-deep_	0.911	0.976	0.899	0.982	0.902
	VGDC	0960	0.993	0.956	0.995	0.955
	CASTOR	0.912	0.985	0.907	0.985	0.909
	COMET	0.864	0.970	0.783	0.976	0.816
**Infl. A (1)**	WalkIm_grayscale-simple_	0.777	0.969	0.771	0.961	0.768
	WalkIm_RGB-simple_	0.756	0.963	0.759	0.955	0.740
	WalkIm_RGB-deep_	0.79	0.974	0.787	0.965	0.785
	VGDC	0.847	0.981	0.845	0.977	0.843
	CASTOR	0.811	0.981	0.817	0.977	0.811
**Infl. A (2)**	WalkIm_grayscale-simple_	0.773	0.971	0.792	0.970	0.768
	WalkIm_RGB-simple_	0.742	0.96	0.7738	0.953	0.72
	WalkIm_RGB-deep_	0.78	0.974	0.781	0.972	0.778
	VGDC	0.849	0.985	0.848	0.979	0.847
	CASTOR	0.803	0.981	0.802	0.977	0.802
**Corona**	WalkIm_grayscale-simple_	0.998	0.999	0.998	0.999	0.998
	WalkIm_RGB-simple_	0.995	0.999	0.995	0.999	0.995
	WalkIm_RGB-deep_	0.984	0.998	0.985	0.996	0.984

**Table 4:** Accuracies of various classification algorithms fed by metabarcoding data set (all methods, except WalkIm, have used 4-mer encoding). Since [37] only reports accuracy of five classification methods and the confusion matrix of DNN, we computed performance metrics of DNN by means of its confusion matrix. Character “-”means that the corresponding measure is not reported by the reference article.

	Se	Sp	PREC	ACC	F1
**WalkIm_grayscale-simple_**	0.945	0.987	0.945	0.978	0.945
**WalkIm_RGB-simple_**	0.966	0.992	0.966	0.987	0.966
**WalkIm_RGB-deep_**	0.945	0.987	0.945	0.978	0.945
**DNN**	0.975	0.989	0.978	0.976	0.976
**SVM**	-	-	-	0.974	-
**K Nearest Neighbors**	-	-	-	0.927	-
**Random Forest**	-	-	-	0.861	-
**XGBoost**	-	-	-	0.972	-

**Table 5:** Classification performance measures achieved by various metagenomics classifiers for four evolutionary levels (i.e. class, order, family, and genus). Character “-”means that the corresponding measure is not reported by the reference article.

			Se	Sp	PREC	ACC	F1
**AMP**	Class	WalkIm_grayscale-simple_	97.43	97.90	97.43	97.72	97.43
		WalkIm_RGB-simple_	98.55	98.85	98.55	98.74	98.55
		WalkIm_RGB-deep_	99.33	99.33	99.33	99.33	99.33
		DLM-CNN	-	-	-	99.41	-
	Order	WalkIm_grayscale-simple_	87.42	98.52	87.24	97.72	87.12
		WalkIm_RGB-simple_	90.34	98.87	90.19	98.28	90.07
		WalkIm_RGB-deep_	94.35	99.40	94.33	99.03	94.31
		DLM-CNN	-	-	-	98.83	-
	Family	WalkIm_grayscale-simple_	76.99	99.12	76.84	98.44	76.67
		WalkIm_RGB-simple_	82.09	99.35	81.93	98.82	81.90
		WalkIm_RGB-deep_	88.30	99.58	88.38	99.24	88.32
		DLM-CNN	-	-	-	97.38	-
	Genus	WalkIm_grayscale-simple_	71.58	98.51	71.70	98.22	71.41
		WalkIm_RGB-simple_	67.29	98.44	67.35	98.11	67.12
		WalkIm_RGB-deep_	89.03	98.65	89.17	98.55	89.05
		DLM-CNN	91.32	-	91.57	91.33	91.18

### Viruses

In Table 3, we compare the WalkIm results for viral data sets with three tolls: a) COMET (as a Markov-based approach customized for HIV and Hepatitis C viruses) [51], b) CASTOR (as a RFLP-based web tool) [3], and c) VGDC tools (as a viral genome data collection tools based on sequence ASCII encoding and CNN) [5]. Of course, as mentioned in the previous section, the results of corona data set are reported separately.

**Fig 7:**
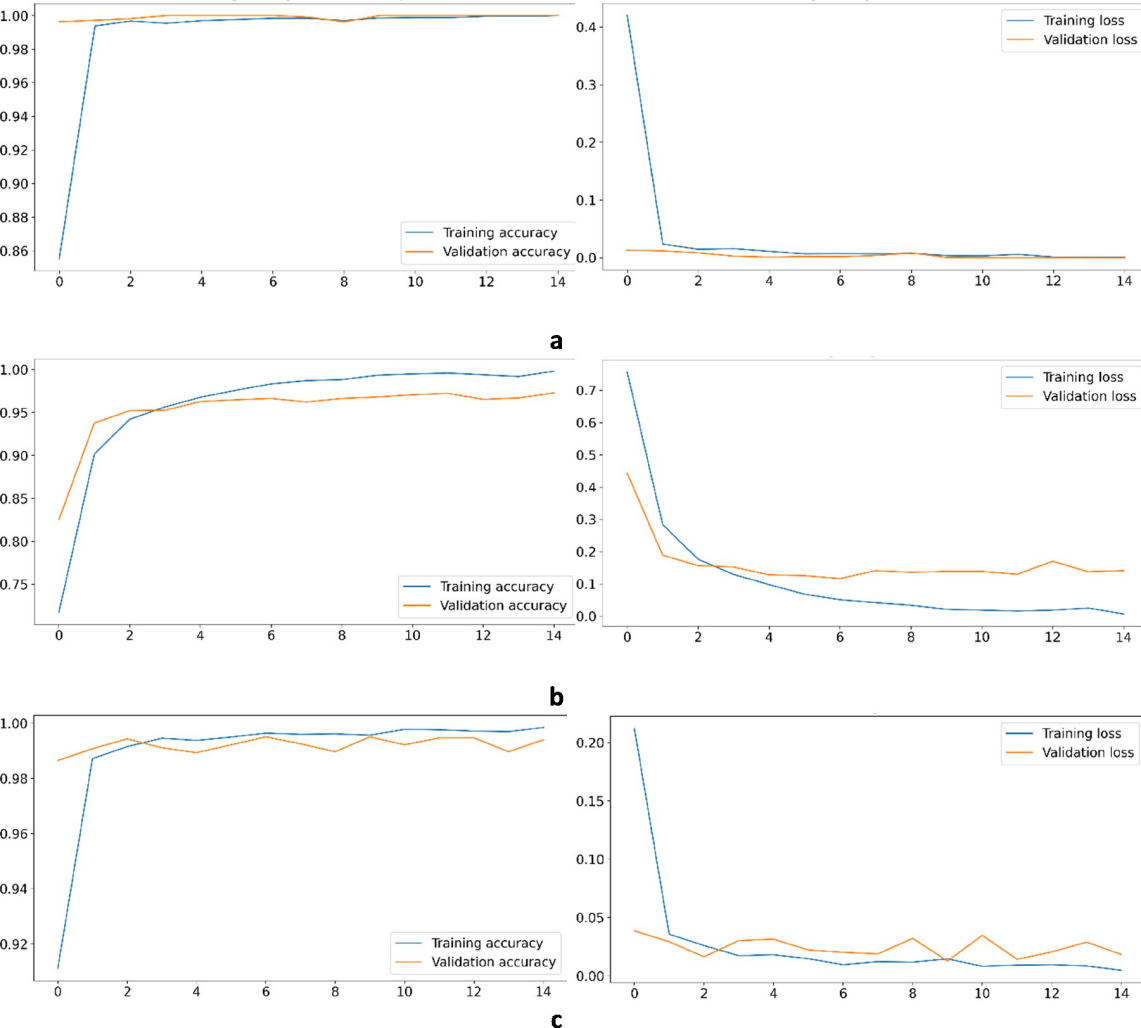
Accuracy and loss convergence diagrams for training and evaluation sets for various epochs; a) Dengue data set, b) metabarcoding data set, and c) AMP class data set.

According to Table 3, the fact that increasing the number of categories reduces the classification accuracy can be deduced in all approaches. It may appear that all formats of WalkIm are less accurate than other approaches, albeit by a modest margin. However, the point is that the CNNs, used in WalkIm, are not customized for any data set, whereas in other approaches they are finely tuned. So, although the difference is not significant, WalkIm classifier would achieve better results if it is customized as well. Especially, for datasets like the influenza A, accuracy improvement is readily achievable by neural network customization. The influenza A genome is made up of eight single strands, as found in each class of the datasets utilized in this study. As a result, any imbalance of each item in these classes, as well as the range of their encoded shapes, makes classification difficult, considering that these sequences, compared to other datasets’ sequences, are shorter and have a higher mutation rate. As a result, compared to alternative datasets, the influenza A genomes are more difficult to categorize, and hence, establishing certain hyperparameters for the network, as addressed in many related studies [5, 6], can facilitates the classification task. Moreover, it should be noted that considering CNN_simple_ performance, it achieves higher accuracy with the grayscale input format, compared to the RGB format. On the other hand, while RGB format scored better (on average) across all data sets (i.e. viruses [5], metabarcoding [37], and metagenomics [6]), we adopt it for evaluating virus database with CNN_complex_. The results show that the RGB format, fed to the CNN_complex_, leads to higher accuracies compared to the other two test modes (RGB and grayscale images fed to the CNN_simple_). Finally, it should be emphasized that the differences in classification accuracies of adopting WalkIm to different networks are so small that can be compensated with minor adjustments, such as increasing the sample size of each class or the test data. As a result of the lesser amount of required data, it can be claimed that grayscale produces an acceptable output. WalkIm images, on the other hand, provide key advantages over other approaches, particularly VGDC [5] as a CNN-based counterpart, as follows:

1. Increased scalability: The length of input sequences has no linear effect on the size of input image. However, since [5] employs integer numbers to encode each nucleotide of the input sequence, increasing the length of input sequence directly affects the network input dimensions.
2. Decreased runtime: For the network fed by WalkIm images, very high convergence is achieved after six or seven epochs, whereas usually 1000 epochs are required for VGDC [5] (of course in some cases results are achieved in 200 epochs). In this regard, runtime can be considerably reduced. To clarify this outperformance, the corresponding diagrams for accuracy convergence, in terms of number of epochs, for training and validation sets of Dengue data set) as an example of viral data sets) is shown in Fig 7A.
3. Decreased input data volume: With the ability to scale WalkIm images, the amount of CNN input data can be reduced. For example, a sequence of 11,000 characters can be encoded in a 32 by 32 matrix with 1024 entries. Of course, this advantage is more evident in data sets with long sequences.

### Metabarcoding

For evaluating capability of WalkIm to encode and classify metabarcoding data set, we compare it against [37] which encodes input data using a K-mer based method, and reports the best ones (4-mers) as the basis for choosing proper classification algorithm. Afterwards, [37] evaluates five classification algorithms (i.e. DNN, SVM, K Nearest Neighbors, Random Forest, and XGBoost), and declares DNN to be the best one. Although not frequent, in [37], a small and unbalanced subset of data is chosen from outside the main data set as the test data; yet, their simulation results are given in Table 4 alongside our method’s results.

As shown in Table 4, although no customization is performed for WalkIm network to classify metabarcoding data, it can obtain higher accuracy, compared to DNN, while other metrics are also extremely close. Since RGB format has better results in most data sets, we have evaluated this format utilizing CNN_complex_. For this purpose, we employed CNN_complex_ on this data, which surprisingly leads to a similar accuracy, compare to CNN_simple_ fed by grayscale format. Thus, for this type of data set, CNN_simple_ with RGB encoding can achieve a good result and no more complex CNN is needed. Finally, we would like to emphasize that our network performs brilliantly in terms of convergence speed during training phase (as shown in Fig 7B), but due to the lack of similar result for DNN, we cannot report the comparison results in this issue.

### Metagenomics

For examining Metagenomics data sets, 16S short-read data are obtained using next-generation sequencing technology amplicon (AMP) which only considers some specified 16S hyper variable regions. The AMP data set is quite targeted, in the sense that it comprises the majority of the information content. As this data set are created by [6], we compare our simulation results against those reported in [6], as shown in Table 5.

[6], like other K-mer based methods, examines different k-mer sizes (i.e. 3 to 7) as well as three classification algorithms (i.e. CNN, DBN and RDP), and so, its best achieved result (i.e. 7-mer with CNN) is chosen for our comparative study. We would like to mention that since [6] focuses on the evolutionary level of the genus, all performance metrics are reported for this level (while for other levels, some metrics are missing). In this regard, we can compare it with our method at the genus level in more details and we cannot accurately compare WakIn to DLM-CNN. Of course, genus level classification is especially important due to its challenging issues of high number of classes and significant similarities among various samples of classes.

As shown in Table 5, the categorization becomes increasingly difficult for both approaches as the number of categories increases. However, by increasing the number of categories, all versions of WalkIm achieves a superior result over DLM-CNN method without requiring any particular customization for this data set. Since RGB format has better results in most data sets, we have included this format in CNN_complex_. For this purpose, we used CNN_complex_ to evaluate RGB encoding without any additional adjustments, and the results were as expected: a significant improvement in all metrics. By providing more information than grayscale encoding and deepening the network for metagenomic data sets, RGB format of WalkIm encoding is projected to produce significantly better outcomes, compared to its counterparts [5, 6, 37]. WalkIm not only improves accuracy and performance, but it also minimizes the size of the input data and speeds up the training process.

It should be noted that there is no report of DLM-CNN with which we can compare the convergence speed of training and validation processes for metagenomics data sets. However, for runtime comparison, we obtained the convergence diagram of this data set considering WalkIm method for sequence encoding, as shown in Fig 7C. These results indicate that the convergence speed is considerably improved, similar to the results achieved for viruses and metabarcoding data sets in Fig 7A and 7B, respectively. The convergence diagrams versus the number of epochs are shown in Fig 7C as an example for these data sets for the AMP class.

### Computation time comparison

As discussed before, optical technologies can considerably speed up CNN architectures. For detailed runtime analysis of optical CNN, we formulate the computational latency and mathematically estimate the runtime of optical CNN utilized for implementation of WalkIm. As shown in S1 Fig in S1 File, considering the conventional optical setup implementing CNN, optical beams pass through each layer of CNN, and hence, each layer adds a certain amount of latency to their path [27]. As a result, total latency (i.e. the time it takes for data to enter and exit a CNN structure) can be calculated by the number of layers within the CNN, and the propagation latency of each layer. In this manner, Eq 6 and Eq 7 formulate the latency of WalkIm’s CNN architecture.

$$Latency_{simpleCNN}=T_{input}+3T_{conv}+3T_{ReLU}+3T_{MP}+T_{camera}+T_{transferData}$$ Eq 6

$$Latency_{deepCNN}=T_{input}+6T_{conv}+6T_{ReLU}+3T_{MP}+T_{camera}+T_{transferData}$$ Eq 7

Where, T_input_ is the time it takes to feed the input image to the network, T_conv_, T_ReLU_, and T_MP_ represent propagation latencies through each optical convolution layer, optical ReLU layer, and optical max pooling layer, respectively, T_camera_ is the time it takes to capture network’s output image by the CCD camera, as the end point of the optical setup, and finally T_transferData_ represent transfer delay of the cable interconnecting CCD camera to the computer system. As obvious, various coefficients in Eq 6 and Eq 7 represent number of corresponding optical layers within the optical setup. For more detailed description, Section "Speed analysis and estimation of optical WalkIm CNN" in S1 File provides more information on this topic.

Considering conventional values for optical latency parameters, as listed in Table 6, we can estimate runtime of the proposed optical structure for both CNNs (i.e. CNN_simple_ and CNN_complex_) as 0.45 ms, taking into account input to output data propagation through the WalkIm optical CNNs. The reason for the lack of time difference between the optical structures of the two CNNs is the negligible propagation delays of the extra optical layers included in CNN_complex_ architecture, compared to CNN_simple_ architecture. In this manner, multiplying the number of input samples in each training set by the calculated "latency" is all that is required to compute the training time. Since 80% (for five-fold assessments) or 90% (for ten-fold assessments) of each data set is usually considered as the training set, the corresponding training time of the optical network can be easily calculated for various architectures, as shown in Table 7.

**Table 6:** Latency estimation for different parts of the free-space optical CNN.

Parameters	Time (s)	Reference
**T_input_**	0.05 m	[27, 52]
**T_conv_**	10 p	[27]
**T_ReLU_**	25 p	[27]
**T_MP_**	10 p	[53]
**T_camera_**	0.4 m	[54]
**T_transferData_**	0.5 μ	Our estimation

**Table 7:** Runtime for training networks in three methods VGDC (viral whole-genome classifier), DLM-CNN_metagenomics_ (metagenomics classifier), and WalkIm (general-purpose sequence classifier with two electrical and optical implementation modes). DNN_metabarcoding_ [37] that classify metabarcoding data did not report the corresponding runtime. Character “-”means that the corresponding measure is not reported by the reference article.

Data sets		Time (s)				
		WalkIm			VGDC	DLM-CNN_metagenomics_
		Electrical		Optical		
		CNN_simple_	CNN_complex_			
**Viral**	Dengue	14	350	13.737	6000 > T > 1200	-
	Hepatitis B (1)	80	2560	18.914	2000 > T > 400	
	Hepatitis B (2)	150	1200	40.234	5000 > T > 1000	
	Hepatitis C	45	22500	10.900	10000 > T > 2000	
	HIV-1 (1)	600	24000	41.457	11000 > T > 2200	
	HIV-1 (2)	700	28000	82.099	11000 > T > 2200	
	Influenza A (1)	12100	96500	1094.298	151000 > T > 30200	
	Influenza A (2)	13200	98000	1108.681	153000 > T > 30600	
	Corona	11	363	3.464	-	
**Metagenomics**	Class	215	5500	56.763	-	24204.754
	Order	940	22000	227.052		
	Family	1250	27500	283.815		
	Genus	1590	33000	340.578		
**Metabarcoding**		180	5100	50.843	-	-

According to Table 7, runtime of DNN_metabarcoding_ is not reported in [37], while the authors only confirm that runtime and memory utilization can be a serious concern during the training process. For the same reason, it is not possible to compare WalkIm method in terms of runtime for metabarcoding data set. On the other hand, since runtime of the training phase for one fold of metagenomics data set is reported in [6], we also provide runtime of the training phase for optical and electrical implementations of WalkIm for one fold.

For a fair comparison of VGDC runtime with that of WalkIm, we should note that VGDC reports the execution time of each epoch [6]. So, we multiply this time by the number of epochs required for training each data set to achieve the training runtime of a fold. As shown in Table 7, training runtimes for VGDC are reported within the ranges of minimum and maximum values, because of the fact that while the maximum number of training epochs for VGDC is 1000, it has been claimed that some data sets had an early stop at about 200 epochs [5]. As can be seen in Table 7, for Hepatitis B (1), Hepatitis C, HIV (1), HIV (2), metagenomics_family_ and metagenomics_ordeer_ data sets, runtime of electrical implementation of WalkIm’s CNN_complex_ exceeds the maximum runtime reported for VGDC. However, it should be noted that when comparing the runtimes of any two methods, the corresponding hardware specifications should be considered as well. Considering this comparative study, it is worth noting that VGDC takes advantages of a more powerful hardware implementation, compare to WalkIm. The detailed hardware specifications for implementing VGDC and WalkIm are listed in Table 8. Moreover, it should be noted that while VGDC runs on a GPU, the electrical version of WalkIm runs on a CPU, taking into account that generally, GPUs can reduce the runtime by, at least, 4 to 5 times [55]. In this manner, we can conclude that even the electrical implementation of WalkIm’s CNN_complex_ is substantially faster than VGDC. And of course, the most crucial point is that, in these data sets, the electrical implementation of CNN_complex_ performed worse, whereas the electrical implementation of CNN_simple_, which performed better, is still faster even with these hardware limitations. It is crystal clear that the optical implementation of WalkIm is much faster than all other electrical counterparts, with a speed up by a factor of 60 to more than 550.

**Table 8:** Hardware specifications of the systems used for simulating WalkIm, VGDC, and DNN_metagenomics_.

WalkIm	VGDC	DNN_metagenomics_
a desktop computer with the following configuration: • CPU: i7-6500 2.5 GHz • RAM: 8 GB and DDR3 2GB • GPU: GeForce GTX 920M	a desktop computer with the following configuration: • CPU: i7-960 3.2 GHz • RAM: 24 GB and DDR5 12 GB • GPU: GeForce GTX TITAN X	a cluster composed of 24 nodes with the following configuration: • CPU: 1 X Intel(R) Xeon(R) CPU E5-2670 0 2.60GHz • RAM: 128 GB Memoria DDR3 1600 MHz • GPU: 48 x GPU NVIDIA KEPLER K20 • HD: 1TB SATA • OS: Centos 6.3

Similar to the comparative study of WalkIm and VGDC for the virus data sets, as follows, we compare training runtimes of WalkIm and DLM-CNN for the metagenomics data set. As shown in Table 7, by increasing the number of categories in the family and genus data sets, the speed of WalkIm reduces compared to DLM-CNN. However, it should be emphasized in [6], execution runtime of DLM-CNN is reported for a cluster of 24 processing nodes (each includes single CPU and 48 GPUs), which is tens of times more powerful than the desktop computer on which WalkIm is executed. Therefore, we can conclude assuming similar hardware specifications, even the electrical implementation of WalkIm considerably outperforms DLM-CNN.

Finally, when it comes to WalkIm’s execution time, it is important to note that the size of the input image influences the training speed. Initially, we used to implement this function with the image size of 256 × 256 pixels. However, we scaled these images down to the smaller sizes to the extent that accuracy is not reduced. As a result, we can simply reduce the input size while increasing the training speed and preserving the accuracy. In addition to speed enhancement, we demonstrated that WalkIm encodes information in a way that scaling cannot affect them. Detailed information on data set scaling can be found in the section "Encoding details" of S1 File.

## Conclusion

WalkIm, our proposed encoding method, focuses on image representation of biological sequences and their usage in Convolutional Neural Networks (CNN). While it is too efficient to be implemented even on the simple desktop systems, it is compatible with free-space optical technology, empowering CNN implementation for big data processing. WalkIm encoding, as a novel extension of DNA-walk encoding, offers various advantages, such as statistical interpretability of the nucleotide distribution, as well as similarity of encoded normal, reversed, and reverse-complemented sequences. Although WalkIm, as a universal method, can be used to classify any sequences based on their DNA and RNA strings, in this paper, we evaluate it by classifying virus sequences (e.g. Coronaviruses, Dengue, HIV, Hepatitis B and C, and Influenza A), metagenomics data, and metabarcoding data. In this study, WalkIm was able to compete with state of the art methods of each field (VGDC [5], COMET [51] and CASTOR [3] for viruses subtyping, DLM-CNN for metagenomics data [6], and [37] for metabarcoding data) in terms of accuracy and training speed without imposing any network adjustments for a specific data set Although tuning-free property of WalkIm facilitates its usage for classifying various data sets with no initialization phase, by proper adjustment of network parameters for each data set, WalkIm can significantly outperform other methods as well. Moreover, WalkIm performance is such that while maintaining accuracy, compared to alternative methods, it can improve the training speed on desktop systems from 1.5 times to 1500 for various data set. We have also shown that taking advantages of free space optical technology for WalkIm implementation, we can improve training speed by more than 400 times, compared to its electrical implementation. It is worth noting that for complex neural networks and large data sets, running WalkIm on a desktop achieves up to 26 times higher speed than alternative methods, like DLM-CNN. Finally, we compared WalkIm with some of the existing fast and accurate methods, such as CASTOR for classification of viruses, where WalkIm reached similar accuracy for various data sets, while CASTOR completed training of 250,000 samples after a few days. Despite all these advantages, WalkIm also faces some challenges. Indeed, although we have evaluated the image’s size and scale for all examined datasets, these parameters must be investigated for other datasets as well. To be able to employ any type of data with the specified parameters, it is required to address the relationship between the image dimensions for WalkIm encoding and the length and type of the sequences in the future works.

## Supporting information

S1 File All supplementary material of this work. This file includes all figures, tables and information mentioned as supplementary material.
(DOCX)

## References

[1] Miladi M. et al., “The landscape of SARS-CoV-2 RNA modifications,” *bioRxiv*, p. 2020.07.18.204362, 2020.

[2] Randhawa G. S., Soltysiak M. P. M., El Roz H., de Souza C. P. E., Hill K. A., and Kari L., “Machine learning using intrinsic genomic signatures for rapid classification of novel pathogens: COVID-19 case study,” *PLoS One*, vol. 15, no. 4, pp. 1–24, 2020. doi: 10.1371/journal.pone.0232391 32330208 PMC7182198

[3] Remita M. A., Halioui A., Malick Diouara A. A., Daigle B., Kiani G., and Diallo A. B., “A machine learning approach for viral genome classification,” *BMC Bioinformatics*, vol. 18, no. 1, p. 208, Dec. 2017. doi: 10.1186/s12859-017-1602-3 28399797 PMC5387389

[4] Solis-Reyes S., Avino M., Poon A., and Kari L., “An open-source k-mer based machine learning tool for fast and accurate subtyping of HIV-1 genomes,” *PLoS One*, vol. 13, no. 11, p. e0206409, Nov. 2018. doi: 10.1371/journal.pone.0206409 30427878 PMC6235296

[5] Fabijanska A. and Grabowski S., “Viral Genome Deep Classifier,” *IEEE Access*, vol. 7, pp. 81297–81307, 2019.

[6] Fiannaca A. et al., “Deep learning models for bacteria taxonomic classification of metagenomic data,” *BMC Bioinformatics*, vol. 19, no. S7, p. 198, Jul. 2018. doi: 10.1186/s12859-018-2182-6 30066629 PMC6069770

[7] Saha S., Johnson J., Pal S., Weinstock G. M., and Rajasekaran S., “MSC: a metagenomic sequence classification algorithm,” *Bioinformatics*, vol. 35, no. 17, pp. 2932–2940, Sep. 2019. doi: 10.1093/bioinformatics/bty1071 30649204 PMC6931357

[8] Randhawa G. S., Hill K. A., Kari L., and Hancock J., “MLDSP-GUI: An alignment-free standalone tool with an interactive graphical user interface for DNA sequence comparison and analysis,” *Bioinformatics*, vol. 36, no. 7, pp. 2258–2259, 2020. doi: 10.1093/bioinformatics/btz918 31834361

[9] Camacho C. et al., “BLAST+: architecture and applications,” *BMC Bioinformatics*, vol. 10, no. 1, p. 421, Dec. 2009. doi: 10.1186/1471-2105-10-421 20003500 PMC2803857

[10] Edgar R. C., “Search and clustering orders of magnitude faster than BLAST,” *Bioinformatics*, vol. 26, no. 19, pp. 2460–2461, Oct. 2010. doi: 10.1093/bioinformatics/btq461 20709691

[11] Alcantara L. C. J. et al., “A standardized framework for accurate, high-throughput genotyping of recombinant and non-recombinant viral sequences,” *Nucleic Acids Res*., vol. 37, no. Web Server, pp. W634–W642, Jul. 2009. doi: 10.1093/nar/gkp455 19483099 PMC2703899

[12] Pineda-Peña A.-C. et al., “Automated subtyping of HIV-1 genetic sequences for clinical and surveillance purposes: Performance evaluation of the new REGA version 3 and seven other tools,” *Infect*. *Genet*. *Evol*., vol. 19, pp. 337–348, Oct. 2013. doi: 10.1016/j.meegid.2013.04.032 23660484

[13] Zielezinski A., Vinga S., Almeida J., and Karlowski W. M., “Alignment-free sequence comparison: benefits, applications, and tools,” *Genome Biol*., vol. 18, no. 1, p. 186, Dec. 2017. doi: 10.1186/s13059-017-1319-7 28974235 PMC5627421

[14] Li Y., He L., Lucy He R., and Yau S. S. T., “A novel fast vector method for genetic sequence comparison,” *Sci*. *Rep*., vol. 7, no. 1, pp. 1–11, 2017. doi: 10.1038/s41598-016-0028-x 28939913 PMC5610321

[15] Lichtblau D., “Alignment-free genomic sequence comparison using FCGR and signal processing,” *BMC Bioinformatics*, vol. 20, no. 1, pp. 1–17, 2019. doi: 10.1186/s12859-018-2565-8 31888438 PMC6937637

[16] Jurtz V. I. et al., “An introduction to deep learning on biological sequence data: examples and solutions,” *Bioinformatics*, vol. 33, no. 22, pp. 3685–3690, Nov. 2017. doi: 10.1093/bioinformatics/btx531 28961695 PMC5870575

[17] Al-Ajlan A. and El Allali A., “CNN-MGP: Convolutional Neural Networks for Metagenomics Gene Prediction,” *Interdiscip*. *Sci*. *Comput*. *Life Sci*., vol. 11, no. 4, pp. 628–635, Dec. 2019. doi: 10.1007/s12539-018-0313-4 30588558 PMC6841655

[18] Morales J. A. et al., “Deep Learning for the Classification of Genomic Signals,” *Math*. *Probl*. *Eng*., vol. 2020, pp. 1–9, May 2020.

[19] Paul T., Vainio S., and Roning J., “Clustering and classification of virus sequence through music communication protocol and wavelet transform,” *Genomics*, vol. 113, no. 1, pp. 778–784, Jan. 2021. doi: 10.1016/j.ygeno.2020.10.009 33069829 PMC7561519

[20] Hoang T., Yin C., and Yau S. S.-T., “Splice sites detection using chaos game representation and neural network,” *Genomics*, vol. 112, no. 2, pp. 1847–1852, Mar. 2020. doi: 10.1016/j.ygeno.2019.10.018 31704313

[21] Deschavanne P. J., Giron A., Vilain J., Fagot G., and Fertil B., “Genomic signature: characterization and classification of species assessed by chaos game representation of sequences,” *Mol*. *Biol*. *Evol*., vol. 16, no. 10, pp. 1391–1399, Oct. 1999. doi: 10.1093/oxfordjournals.molbev.a026048 10563018

[22] Liang Q., Bible P. W., Liu Y., Zou B., and Wei L., “DeepMicrobes: taxonomic classification for metagenomics with deep learning,” *NAR Genomics Bioinforma*., vol. 2, no. 1, Mar. 2020. doi: 10.1093/nargab/lqaa009 33575556 PMC7671387

[23] Fonseca V. et al., “A computational method for the identification of Dengue, Zika and Chikungunya virus species and genotypes,” *PLoS Negl*. *Trop*. *Dis*., vol. 13, no. 5, p. e0007231, May 2019. doi: 10.1371/journal.pntd.0007231 31067235 PMC6527240

[24] De Marinis L., Cococcioni M., Castoldi P., and Andriolli N., “Photonic Neural Networks: A Survey,” *IEEE Access*, vol. 7, pp. 175827–175841, 2019.

[25] Xu S., Wang J., Wang R., Chen J., and Zou W., “High-accuracy optical convolution unit architecture for convolutional neural networks by cascaded acousto-optical modulator arrays,” *Opt*. *Express*, vol. 27, no. 14, p. 19778, Jul. 2019. doi: 10.1364/OE.27.019778 31503733

[26] Yan T. et al., “Fourier-space Diffractive Deep Neural Network,” *Phys*. *Rev*. *Lett*., vol. 123, no. 2, p. 023901, Jul. 2019. doi: 10.1103/PhysRevLett.123.023901 31386516

[27] Colburn S., Chu Y., Shilzerman E., and Majumdar A., “Optical frontend for a convolutional neural network,” *Appl*. *Opt*., vol. 58, no. 12, p. 3179, Apr. 2019. doi: 10.1364/AO.58.003179 31044792

[28] Jiao S., Feng J., Gao Y., Lei T., Xie Z., and Yuan X., “Optical machine learning with incoherent light and a single-pixel detector,” *Opt*. *Lett*., vol. 44, no. 21, p. 5186, Nov. 2019. doi: 10.1364/OL.44.005186 31674963

[29] Chang J., Sitzmann V., Dun X., Heidrich W., and Wetzstein G., “Hybrid optical-electronic convolutional neural networks with optimized diffractive optics for image classification,” *Sci*. *Rep*., vol. 8, no. 1, p. 12324, Dec. 2018. doi: 10.1038/s41598-018-30619-y 30120316 PMC6098044

[30] Sievers A. et al., “K-mer content, correlation, and position analysis of genome dna sequences for the identification of function and evolutionary features,” *Genes (Basel)*., vol. 8, no. 4, pp. 1–18, 2017. 10.3390/genes8040122 PMC5406869 28422050

[31] “Survey on encoding schemes for genomic data representation and feature learning—from signal processing to machine learning,” *Big Data Min*. *Anal*., vol. 1, no. 3, pp. 191–210, Sep. 2018.

[32] Hewelt B., Li H., Jolly M. K., Kulkarni P., Mambetsariev I., and Salgia R., “The DNA walk and its demonstration of deterministic chaos—relevance to genomic alterations in lung cancer,” *Bioinformatics*, vol. 35, no. 16, pp. 2738–2748, Aug. 2019. doi: 10.1093/bioinformatics/bty1021 30615123 PMC6691335

[33] Chen Z. et al., “iFeature: a Python package and web server for features extraction and selection from protein and peptide sequences,” *Bioinformatics*, vol. 34, no. 14, pp. 2499–2502, Jul. 2018. doi: 10.1093/bioinformatics/bty140 29528364 PMC6658705

[34] Bonidia R. P., Domingues D. S., Sanches D. S., and de Carvalho A. C. P. L. F., “MathFeature: feature extraction package for DNA, RNA and protein sequences based on mathematical descriptors,” *Brief*. *Bioinform*., Nov. 2021. 10.1093/bib/bbab434 PMC8769707 34750626

[35] Kobori Y. and Mizuta S., “Similarity Estimation Between DNA Sequences Based on Local Pattern Histograms of Binary Images,” *Genomics*, *Proteomics Bioinforma*., vol. 14, no. 2, pp. 103–112, 2016. doi: 10.1016/j.gpb.2015.09.007 27132143 PMC4880953

[36] Liao B., “A 2D graphical representation of DNA sequence,” *Chem*. *Phys*. *Lett*., vol. 401, no. 1–3, pp. 196–199, Jan. 2005.

[37] Nugent C. M. and Adamowicz S. J., “Alignment-free classification of COI DNA barcode data with the Python package Alfie,” *Metabarcoding and Metagenomics*, vol. 4, Sep. 2020.

[38] N. A., “A new graphical representation and analysis of DNA sequence structure. I: Methodology and application to globin genes,” *Curr*. *Sci*., vol. 66, pp. 309–314, 1994.

[39] Qi Y., Jin N., and Ai D., “Wavelet Analysis of DNA Walks on the Human and Chimpanzee MAGE/CSAG-palindromes,” *Genomics*. *Proteomics Bioinformatics*, vol. 10, no. 4, pp. 230–236, Aug. 2012. doi: 10.1016/j.gpb.2012.07.004 23084779 PMC5054716

[40] Zhang Z.-J., “DV-Curve: a novel intuitive tool for visualizing and analyzing DNA sequences,” *Bioinformatics*, vol. 25, no. 9, pp. 1112–1117, May 2009. doi: 10.1093/bioinformatics/btp130 19276149

[41] Maleki E., Babashah H., Koohi S., and Kavehvash Z., “All-optical DNA variant discovery utilizing extended DV-curve-based wavelength modulation,” *J*. *Opt*. *Soc*. *Am*. *A*, vol. 35, no. 11, p. 1929, 2018. doi: 10.1364/JOSAA.35.001929 30461853

[42] Lecun Y., Bottou L., Bengio Y., and Haffner P., “Gradient-based learning applied to document recognition,” *Proc*. *IEEE*, vol. 86, no. 11, pp. 2278–2324, 1998.

[43] Krizhevsky A., Sutskever I., and Hinton G. E., “ImageNet classification with deep convolutional neural networks,” *Commun*. *ACM*, vol. 60, no. 6, pp. 84–90, May 2017.

[44] Szegedy C. et al., “Going deeper with convolutions,” in *2015 IEEE Conference on Computer Vision and Pattern Recognition (CVPR)*, 2015, pp. 1–9.

[45] He K., Zhang X., Ren S., and Sun J., “Deep Residual Learning for Image Recognition,” in *2016 IEEE Conference on Computer Vision and Pattern Recognition (CVPR)*, 2016, pp. 770–778.

[46] Karen S. and Andrew Z., “Very Deep Convolutional Networks for Large-Scale Image Recognition,” *CoRR*, vol. abs/1409.1, 2015.

[47] Akbari Rokn Abadi S., Hashemi Dijujin N., and Koohi S., “Optical pattern generator for efficient bio-data encoding in a photonic sequence comparison architecture,” *PLoS One*, vol. 16, no. 1, p. e0245095, Jan. 2021. doi: 10.1371/journal.pone.0245095 33449928 PMC7810328

[48] Kwan H. K., Kwan B. Y. M., and Kwan J. Y. Y., “Novel methodologies for spectral classification of exon and intron sequences,” *EURASIP J*. *Adv*. *Signal Process*., vol. 2012, no. 1, p. 50, Dec. 2012.

[49] Sergey I. and Christian S., “Batch Normalization: Accelerating Deep Network Training by Reducing Internal Covariate Shift,” in *Proceedings of the 32nd International Conference on Machine Learning*, 2015, pp. 448–456.

[50] Kingma D. P. and Jimmy B., “Adam: A Method for Stochastic Optimization,” *CoRR*, vol. abs/1412.6, 2015.

[51] Struck D., Lawyer G., Ternes A.-M., Schmit J.-C., and Bercoff D. P., “COMET: adaptive context-based modeling for ultrafast HIV-1 subtype identification,” *Nucleic Acids Res*., vol. 42, no. 18, pp. e144–e144, Oct. 2014. doi: 10.1093/nar/gku739 25120265 PMC4191385

[52] Spall J., Guo X., Barrett T. D., and Lvovsky A. I., “Fully reconfigurable coherent optical vector–matrix multiplication,” *Opt*. *Lett*., vol. 45, no. 20, p. 5752, Oct. 2020. doi: 10.1364/OL.401675 33057276

[53] Ayachi R., Afif M., Said Y., and Atri M., “Strided Convolution Instead of Max Pooling for Memory Efficiency of Convolutional Neural Networks,” 2020, pp. 234–243.

[54] “HS7: Fastec Imaging’s HS Series high-speed camera,” *4* october 2021. [Online]. Available: https://hsi.ca/product/hs7/.

[55] BUBER E. and DIRI B., “Performance Analysis and CPU vs GPU Comparison for Deep Learning,” in 2018 6th *International Conference on Control Engineering* & Information Technology (CEIT), 2018, pp. 1–6.

