# Supplementary material for: WalkIm: Compact image-based encoding for high-performance classification of biological sequences using simple tuning-free CNNs
Source: PLoS One. 2022 Apr 15;17(4):e0267106. doi: 10.1371/journal.pone.0267106 (PMC9012348; doi:10.1371/journal.pone.0267106)
Supplement: S1 File — This file includes all figures, tables and information mentioned as supplementary material. (DOCX) [file pone.0267106.s001.docx]

**Supplementary material of**

**WalkIm: compact image-based encoding for high-performance classification of biological sequences using simple tuning-free CNNs**

**Saeedeh Akbari Rokn Abadi^1^ – Amirhossein Mohammadi^1^ – Somayyeh Koohi^1,*^**

**^1^ Department of Computer Engineering, Sharif University of Technology, Tehran, Iran**

**^*^ Correspondence: E-mail: koohi@sharfi.edu**

# Data

Three types of datasets were employed in this study, each of which was retrieved from one of three articles, [1], [2], and [3]. Each category's access information is listed below.

## Viral genome data sets

Each of viral genome datasets has been collected and used from a database, whose search criteria and download links are listed in S1 Table. It should be mentioned that these datasets are introduced in [1], and we downloaded them at 12 December 2020.

**S1 Table** Search options for retrieving genomic sequences [1]

| Virus type | Database | Query options | Download link |
| --- | --- | --- | --- |
| Dengue | NCBI | Sequence type: nucleotide, full-length sequences only, collapse identical sequences, other options: default none | https://www.ncbi.nlm.nih.gov/genomes/VirusVariation/Database/nphselect.cgi?taxid=12637 |
| Hepatitis B | HBVdb | none | https://hbvdb.ibcp.fr/HBVdb/HBVdbDataset?seqtype=0 |
| Hepatitis C | LANL | Genomic region: complete genome, exclude recombinants, exclude problematic, exclude no genotype, other options: default | https://hcv.lanl.gov/components/sequence/HCV/search/searchi.html |
| HIV-1 | LANL | Virus: HIV-1, genomic region: complete genome, subtype: any subtype, excluding problematic, other options default | https://www.hiv.lanl.gov/components/sequence/HIV/search/search.html |
| Influenza A | NCBI | Sequence type: nucleotide, type: A, full-length only, collapse identical sequences, other options: default | https://www.ncbi.nlm.nih.gov/genomes/FLU/Database/nphselect.cgi#mainform |

## Coronaviruses data set

All samples are available at <https://github.com/SAkbari93/WalkIm.git>

## Metabarcoding data set

Among the datasets used to evaluate WalkIm, metabarcoding data set is obtained from []. This dataset is a barcoding dataset consisting of cytochrome c oxidase subunit I (COI) DNA barcode sequences to taxonomic kingdoms, and can be accessed from <https://github.com/CNuge/data-alfie>.

## Metagenomics data sets

We used the data generated by [] for the metagenomics datasets. This bacteria taxonomy consists of four tests for each SG (Shotgun) and AMP (Amplicon) sequencing technologies to classify the same samples into four levels of evolution (i.e. class, order, family, and genus). These samples are accessible from <https://github.com/IcarPA-TBlab/MetagenomicDC/tree/master/data>.

# Optical CNN setup

CNN consists of several layers that can be divided into three essential parts: convolution layers, ReLU layers, and pooling layers. As discussed in [4][5], these CNN structures can be efficiently implemented by optical technology to speed up the comparison procedure. S1 Fig. depicts an optical architecture implementing a general convolutional neural network. Since the optical structures of the convolution layer and the pooling layer are similar, as follows, we will deal with the optical structures of the convolution layer and the ReLU layer.


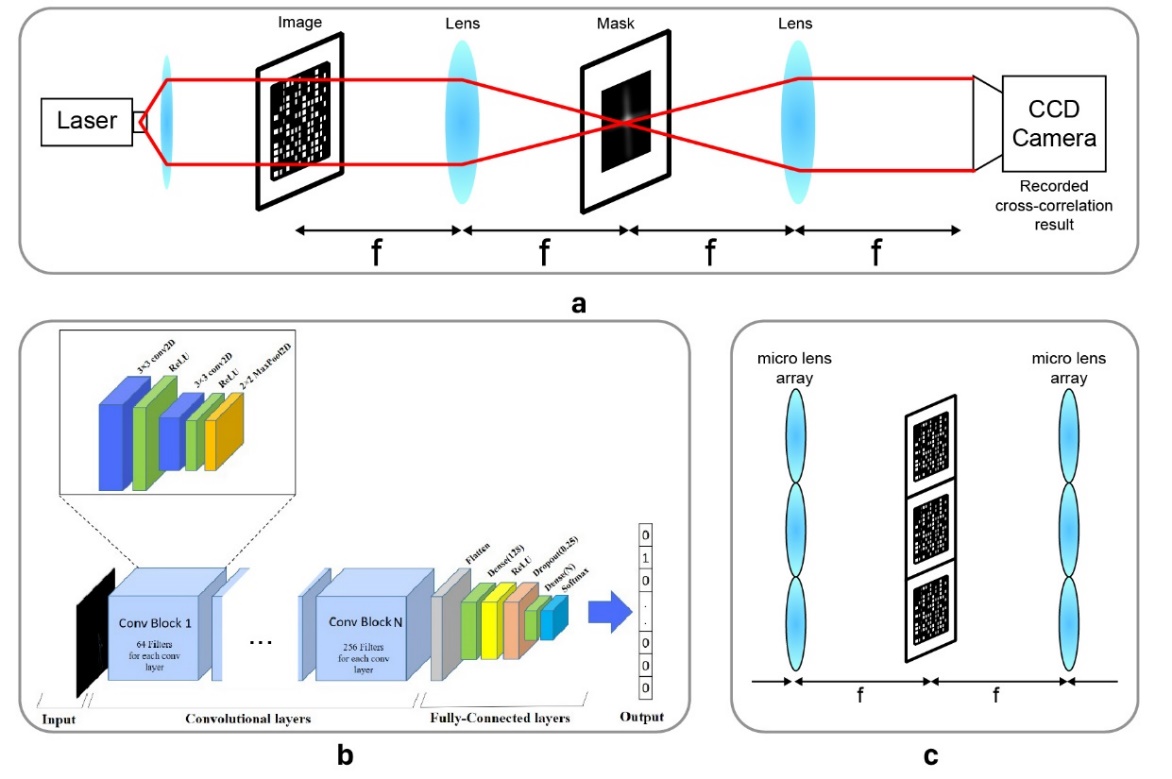


**S1 Fig. Architecture of ONN**: a) Schematic of a single 4f correlator system. The mask is determined by Fourier transform of Kernels from the convolutional layer; b) The schematic architecture of a typical CNN; c) A convolutional layer can be implemented using optical elements, where a lenslet array is used to perform several convolutions in parallel. [4]

## Optical cross-correlation operation

The Fourier transformation property of lenses and filters can be used to achieve optical cross-correlation in the frequency domain. The Fourier transform of the entire 2D pattern would be captured in real-time through a lens, taking advantages of inherent parallel processing capability of optics. It should be noted that the optical configurations of 1D and 2D correlators based on the well-known Vander lugt arrangement [6] are similar, with the exception of lens types (i.e., 1D correlator needs cylindrical lenses [7]). Fig. S 2 depicts the overall structure of the optical setup of 2D cross-correlator, called as 4f setup. Two SLMs (Spatial Light Modulator), SLM_ref_ and SLM_q_, are required for generating encoded reference and query sequences. As shown in this figure, FT of the encoded reference sequence and the encoded query sequence are produced by SLM_ref_ and SLM_q,_ respectively. Assuming constant reference sequence, without loss of generality, FFT of reference sequence can be computed by computer and feed the SLM, which can avoid additional optical Fourier setup.

Optical computations are performed by passing the light originated from the laser source through the SLMs and passive components as follows. SLM_q_ is first illuminated by a coherent light source, whose generated image passes through the lens L1. The FT of q (sequence data encoded by SLM_q_) is computed by the lens L1 and is projected on the SLM_ref_, lcate3d at the focal distance of L1. SLM_ref_, on the other hand, contains R (Fourier transform of ref); therefore, the Dot Product of Q and R is formed immediately at SLM_ref_. Finally, the inverse FT of the product pattern of R.Q can be achieved by light traversal through lens L2. In this manner, CCD camera located at the focal distance of L2 (CCD_detector_ in S2 *Fig.*) determines the cross-correlation of query and reference sequences. S2 *Fig.* depicts all aforementioned steps for computation of 2D optical cross-correlator.


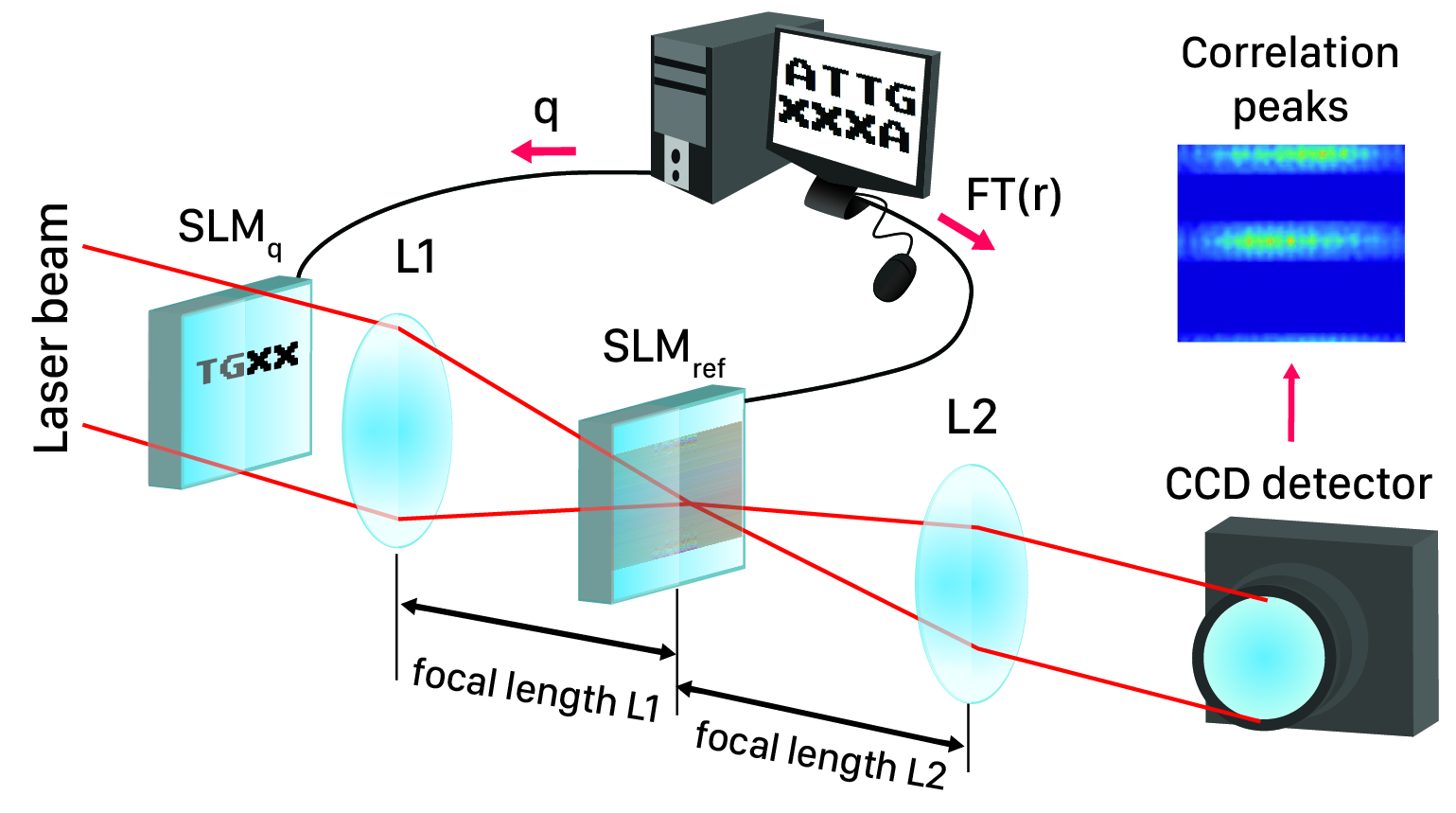


**S2 Fig. Optical setup of 2D cross-correlator** for reference sequence “ATTGCCCA” and query sequence “TGCC” [5]

## Optical nonlinear activation function

The importance of the nonlinear activation function in deep neural networks is very clear. However, the lack of proper optical nonlinear function limits the design of ONNs [4], so it has become a contentious topic in ONNs in recent years [8]. The use of saturable absorption nonlinearity for implementing optical nonlinear activation function is proposed to realize implementation of ONNs. This concept was first presented and designed in [9]. Saturable absorption is an all-optical phenomenon in which light absorption reduces as the amount of light traveling through an atomic vapor increases. It is worth noting that SA's nonlinear functionality is only available for particular input ranges; otherwise, the signal is transformed linearly from input to output.

# Speed analysis and estimation of optical WalkIm CNN

In this section, we discuss computation time of each layer to estimate total processing time of the neural network, due to the fact that different optical neural networks can be constructed by concatenating different layers. Therefore, sum of estimated values for various layers (as listed in **S2** Table) is reported as the estimated processing time of ONN. In this way, we can also estimate the training time of an ONN based on its layers and input data properties.

**S2 Table** estimated processing time of each layer of an ONN

| Switch time among input images (T_input_) | 0.05 m | [4][10] |
| --- | --- | --- |
| Processing time of convolution layer (T_conv_) | 10 p | [4] |
| Processing time of SA layer (T_ReLU_) | 25 p | [4] |
| Processing time of Max-pooling layer (T_MP_) | 10 p | [11] |
| Time required to capture and record the output image by the CCD camera (T_camera_) | 0.4 m | [12] |
| Data transfer time from the camera to the computer system (T_transferData_) | 0.5 μ | Our estimation |

There are various display technologies for generating proper input images for the free-space optical systems. SLM (Spatial Light Modulator) and DMD (Digital micro-Mirror Device) are two well-known equipment utilized in ONN SLMs with a refresh rate of 1 kHz [4] and proportionate commercial DMDs with a refresh rate of 20 kHz [10] can be used in a free-space optical architecture, resulting in 1 ms [4] and 0.05 ms delay values, respectively, for generating an input image (T_input_).

Convolutional and Max-pooling layers can be implemented with 4f structure [11], so the processing time for both layers (i.e. T_conv_ and T_MP_) can be similarly estimated. The lens array implementing optical convolution operations within the convolutional layer is 0.31 cm^2^ in size, as specified in [6], using lenses with diameters and focal lengths of 0.57 mm and 3 mm, respectively. Light signals can pass through the both aforementioned 4f optical structures in a minimal duration of 10 ps [6], thanks to the use of tiny metasurfaces with diameters and focal lengths of 0.57 mm and 3 mm, respectively. Based on the proposed models for SA nonlinear layer [6], the light transmission latency through SA layers, T_ReLU_, can be estimated at about ~25 ps seconds. T_camera_ can be computed as the time it takes for image sensors to capture the output image of an ONN and convert it to the digital domain. Using high-speed commercial cameras [44] with a frame rate of 2500 frames per second, the camera's latency can be estimated to be 0.4 milliseconds. Finally, T_transferData_ data is the time it takes for the digital image to be transmitted via an available communication link, such as USB 3.1 Gen2 with the frame rate of 10 Gbit/s. We can easily compute T_transferData_ as 0.5 us, because the image size in WalkIm encoding is roughly 0.6 kB.

# Encoding details

**S3 Table** image scale of each data set

| Data set | Initial image size | Final image size |
| --- | --- | --- |
| Covid | 1024 x 1024 | 64 x 64 |
| Dengue | 256 x 256 | 32 x 32 |
| Hepatitis B 1 | 256 x 256 | 64 x 64 |
| Hepatitis B 2 | 256 x 256 | 32 x 32 |
| Hepatitis C | 2048 x 2048 | 256 x 256 |
| HIV-1 1 | 2048 x 2048 | 128 x 128 |
| HIV-1 2 | 2048 x 2048 | 128 x 128 |
| Influenza A 1 | 256 x 256 | 64 x 64 |
| Influenza A 2 | 256 x 256 | 64 x 64 |
| Barcoding | 128 x 128 | 64 x 64 |
| AMP class | 128 x 128 | 64 x 64 |
| AMP order | 128 x 128 | 64 x 64 |
| AMP family | 128 x 128 | 64 x 64 |
| AMP Genus | 128 x 128 | 64 x 64 |

# Confusion matrices

As follows, we report the confusion matrices corresponding to the RGB images format tested by CNN_complex_. The confusion matrices of influenza A (1), influenza A (2), and AMP genus data sets are not included in this section due to their extra-large sizes. In the case of your interest, please send us an email to request them.

## Viruses


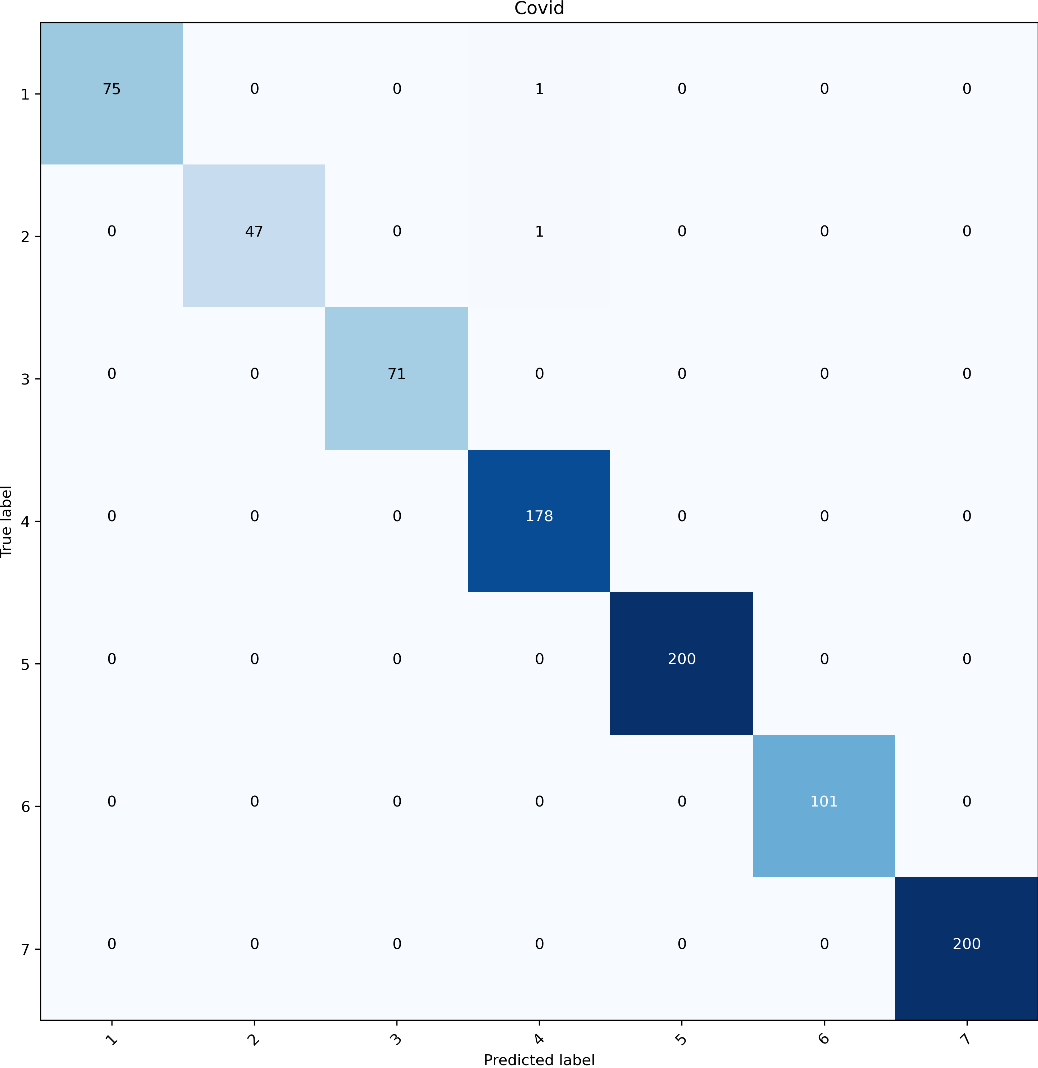


**S3 Fig. Confusion matrix of Coronaviruses data set**


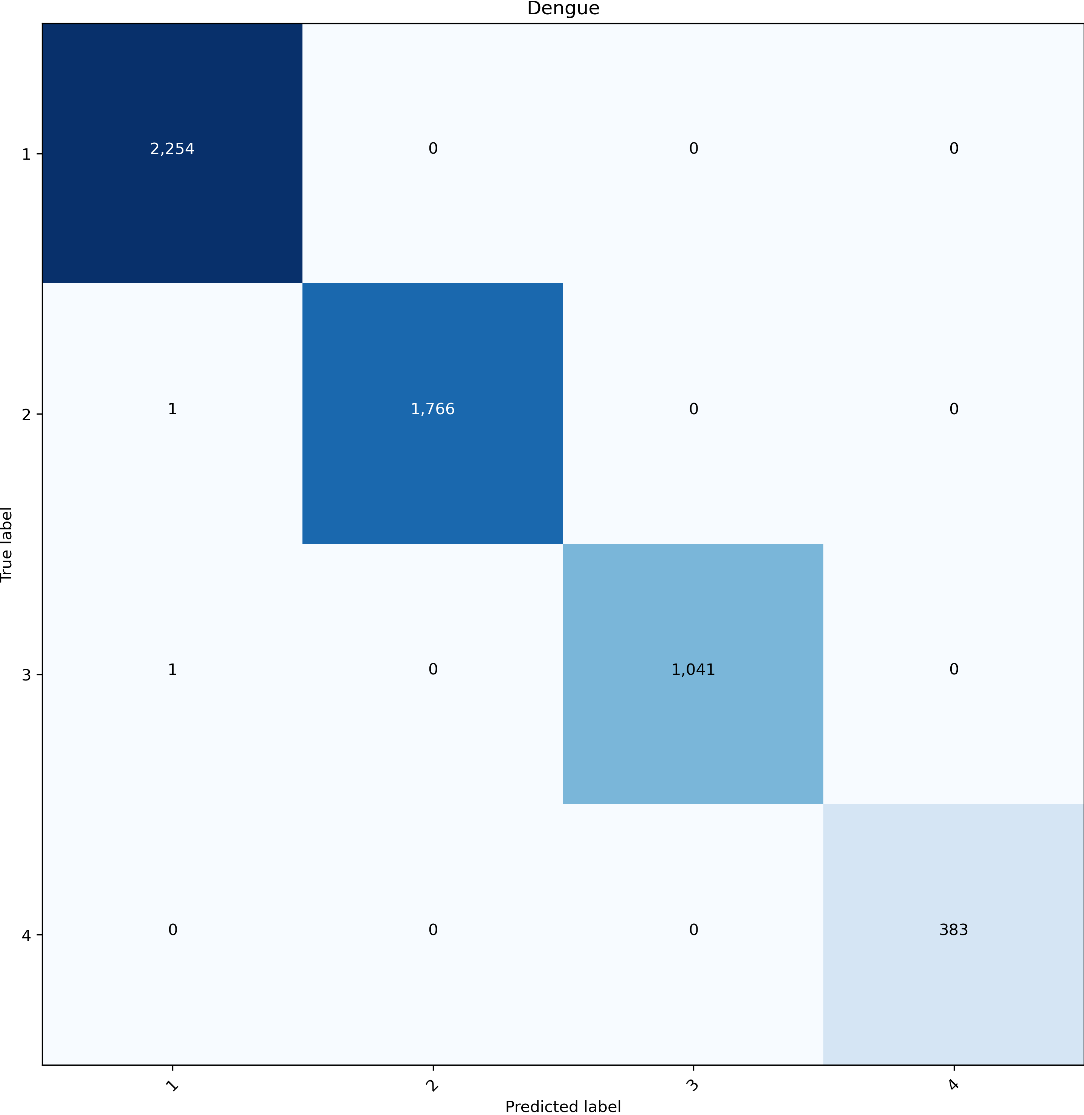


**S4 Fig. Confusion matrix of dengue data set**


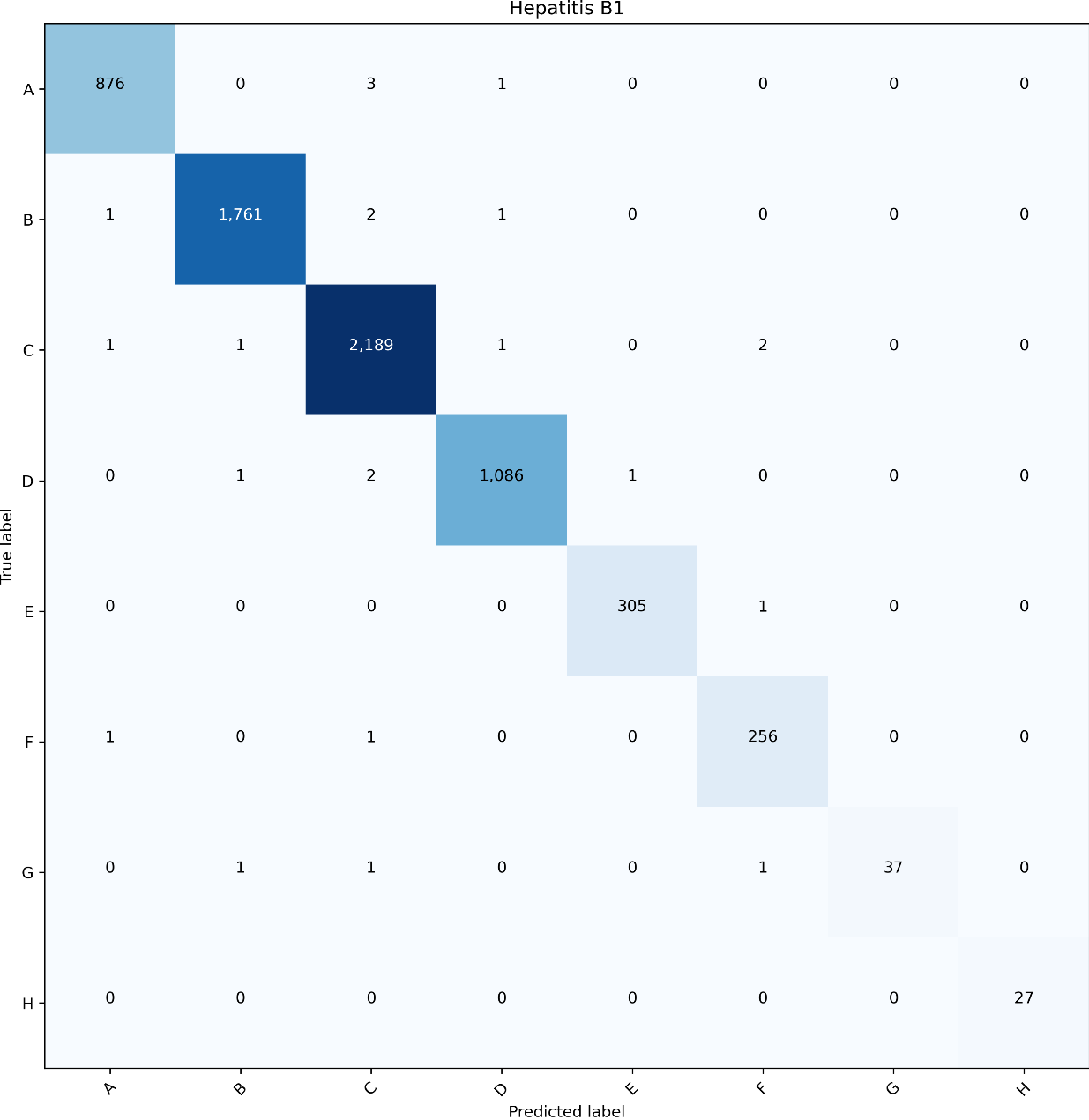


**S5 Fig. Confusion matrix of Hepatitis B (1) data set**


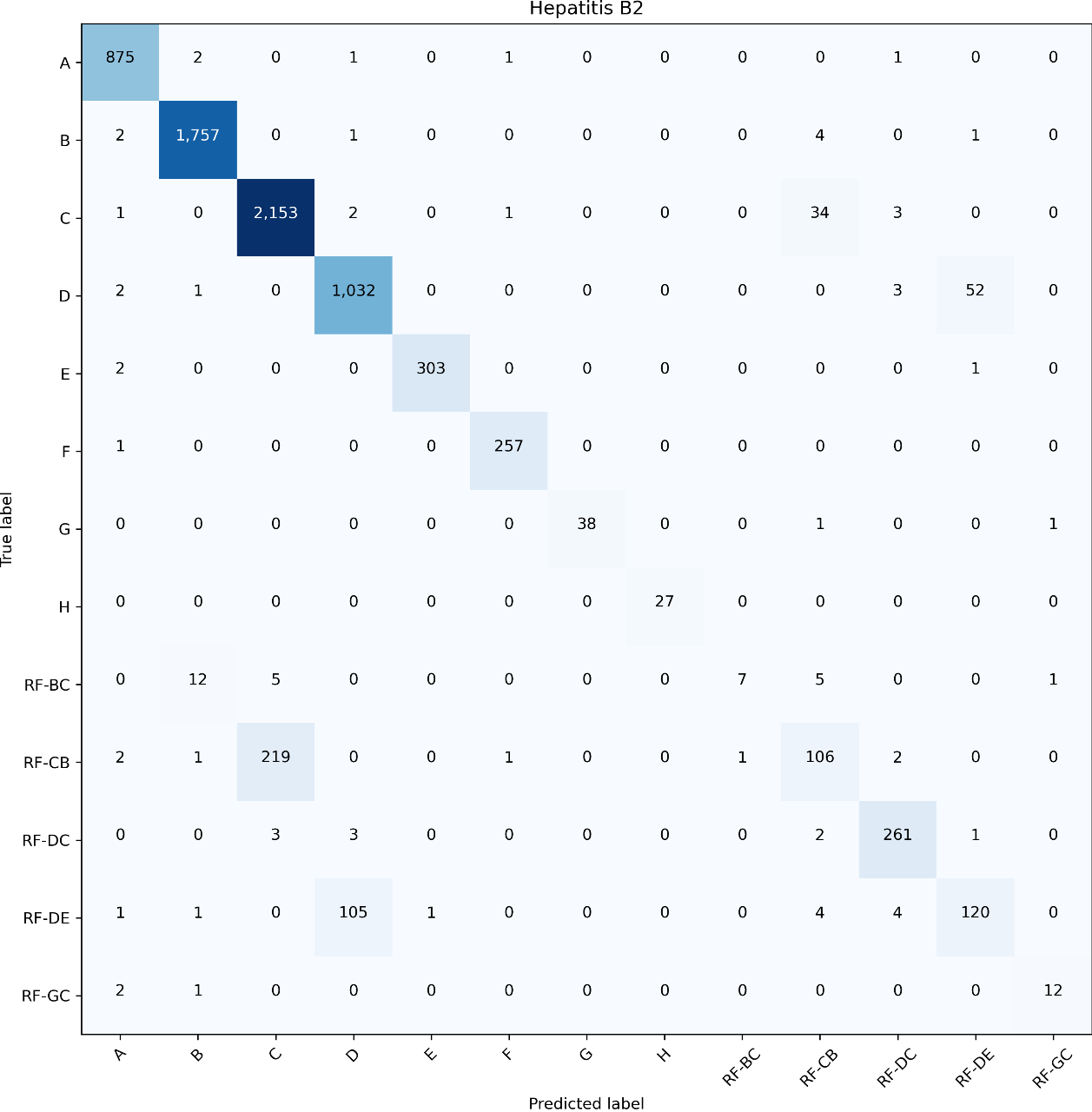


**S6 Fig. Confusion matrix of Hepatitis B (2) data set**


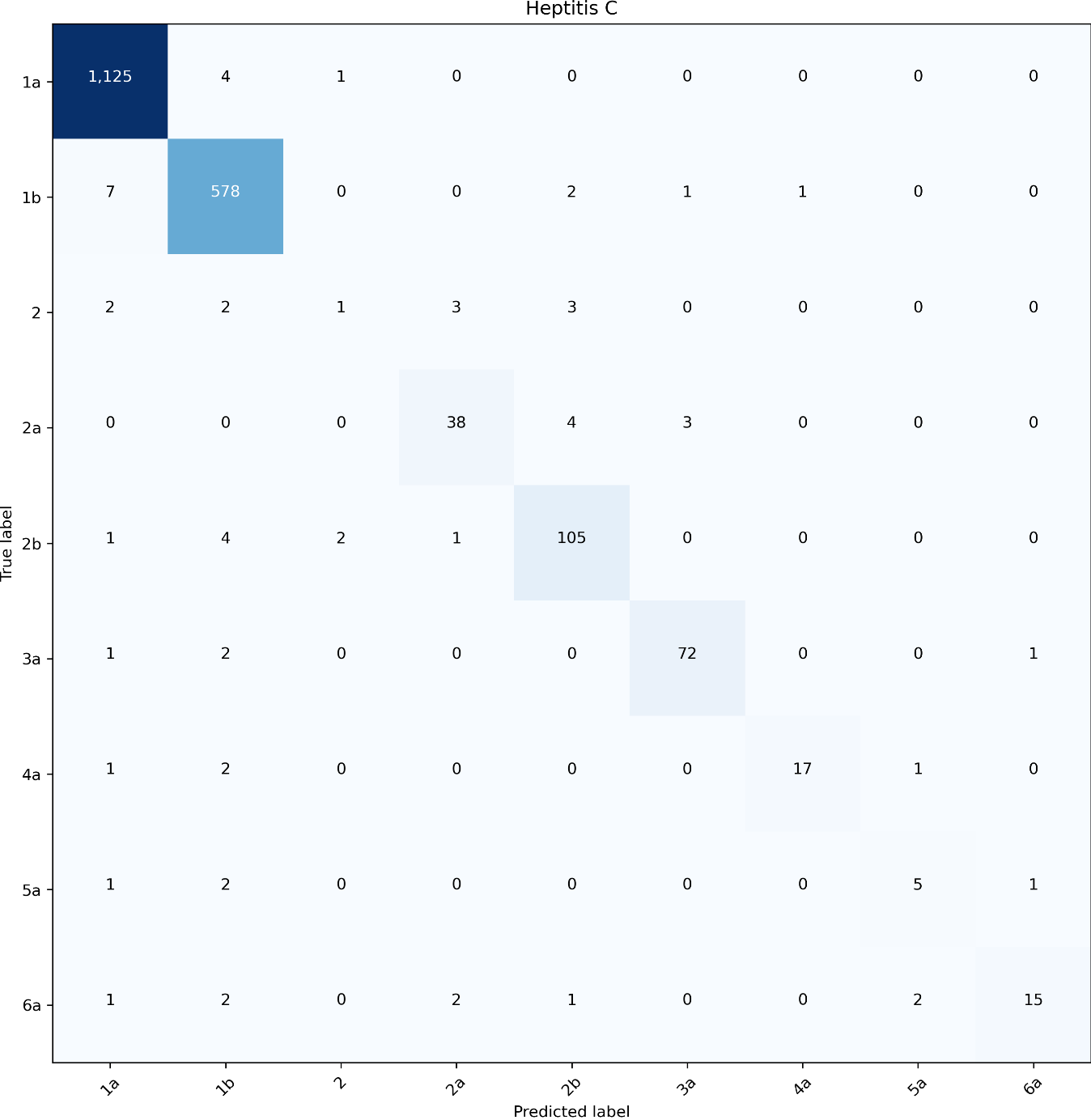


**S7 Fig. Confusion matrix of Hepatitis C data set**


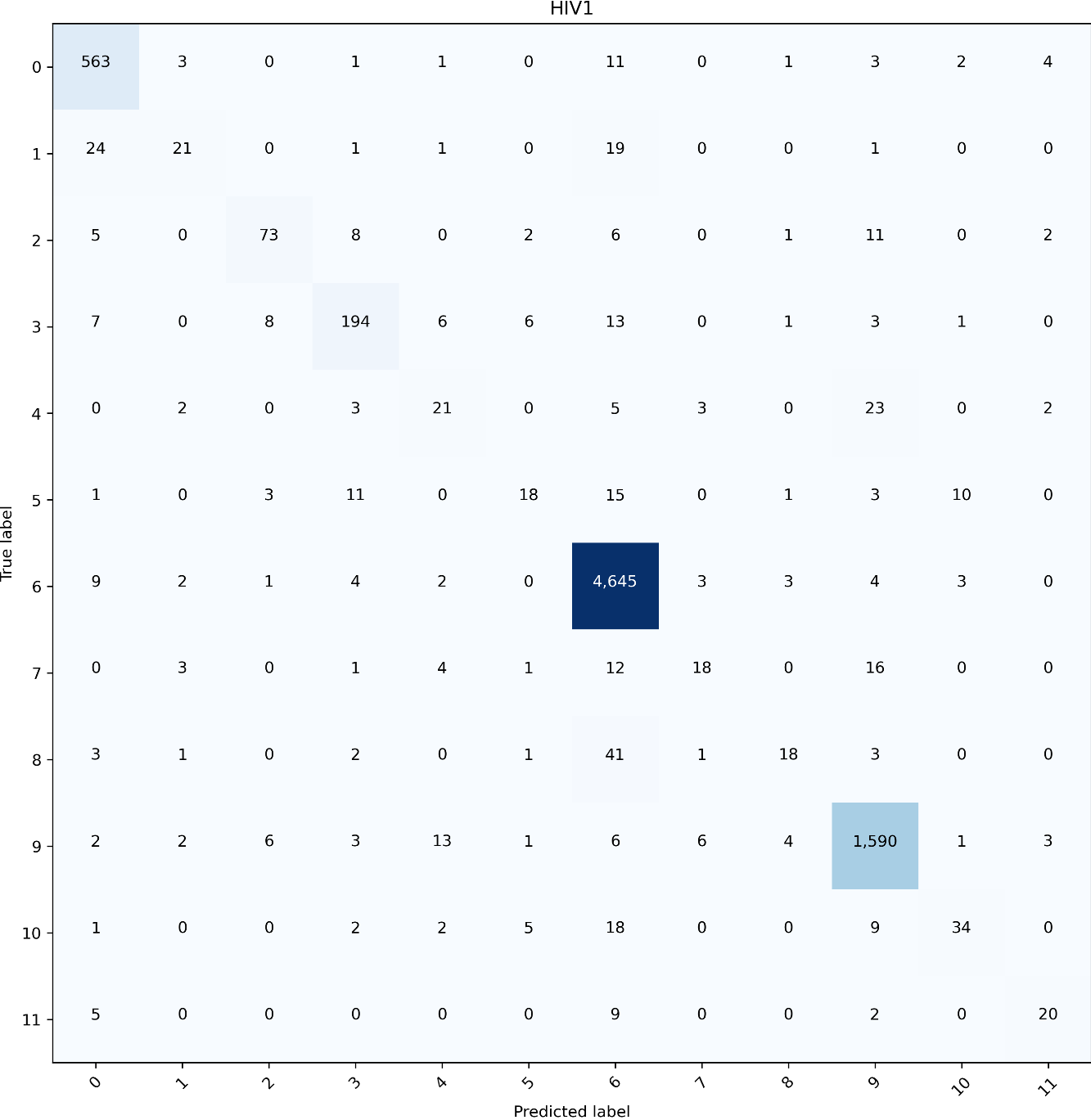


**S8 Fig. Confusion matrix of HIV (1) data set**


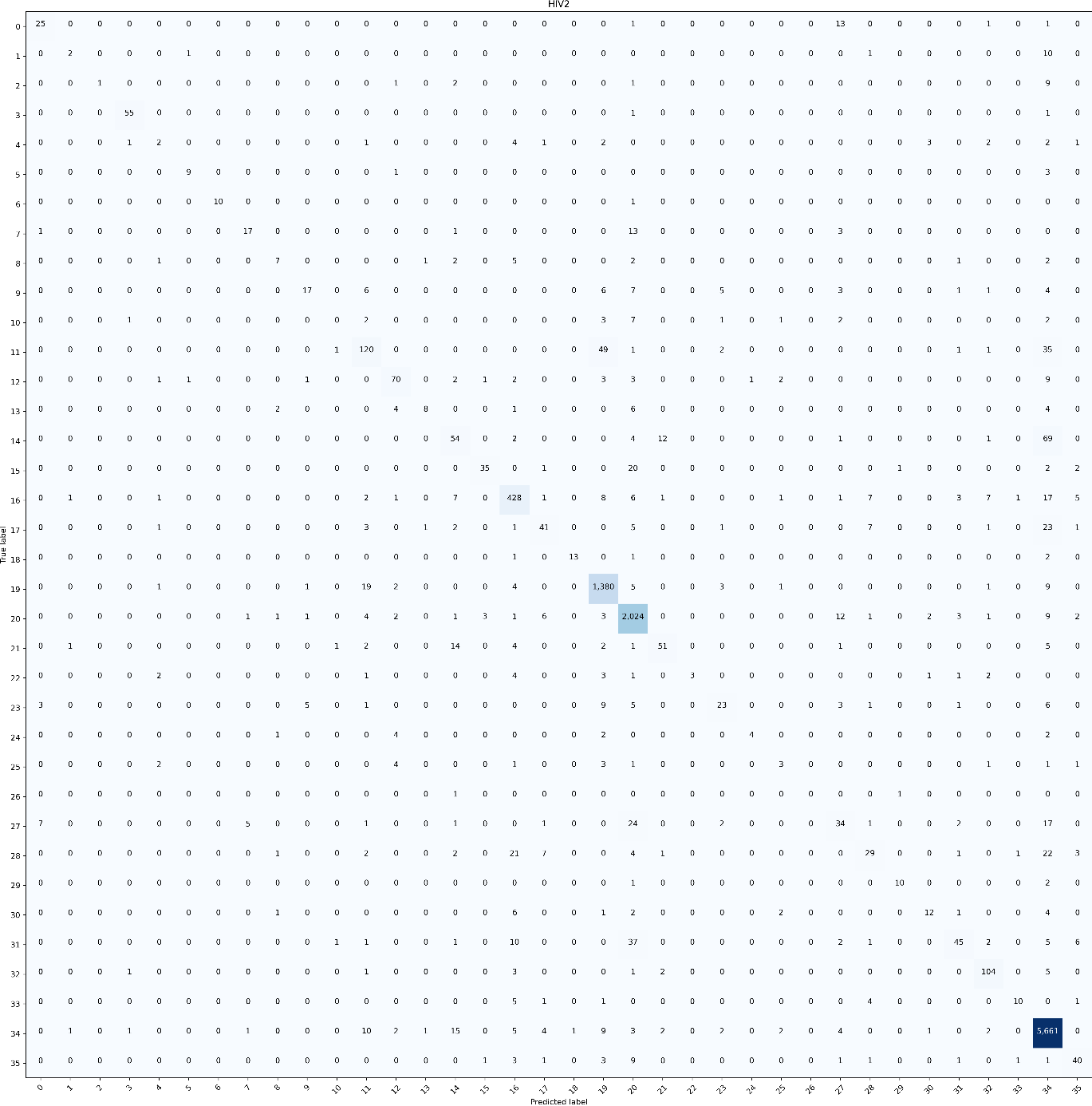


**S9 Fig. Confusion matrix of HIV (2) data set**

## Metabarcoding


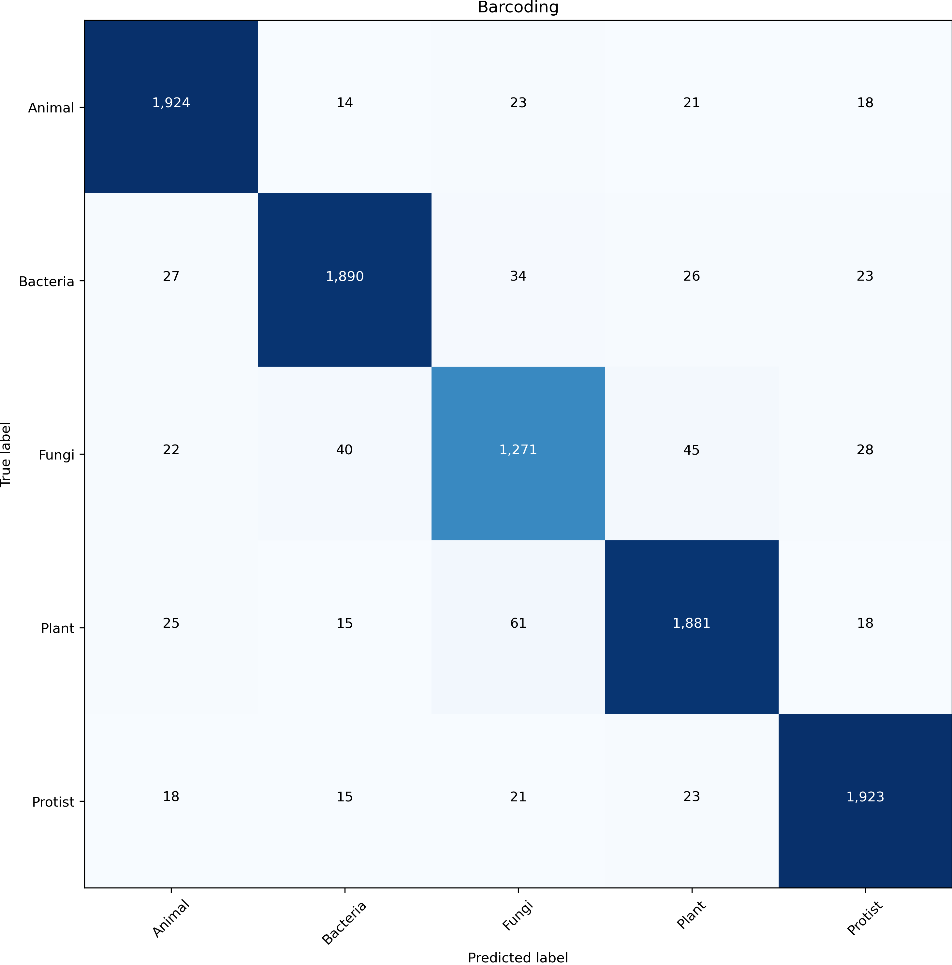


**S10 Fig. Confusion matrix of metabarcoding data set**

## Metagenomics


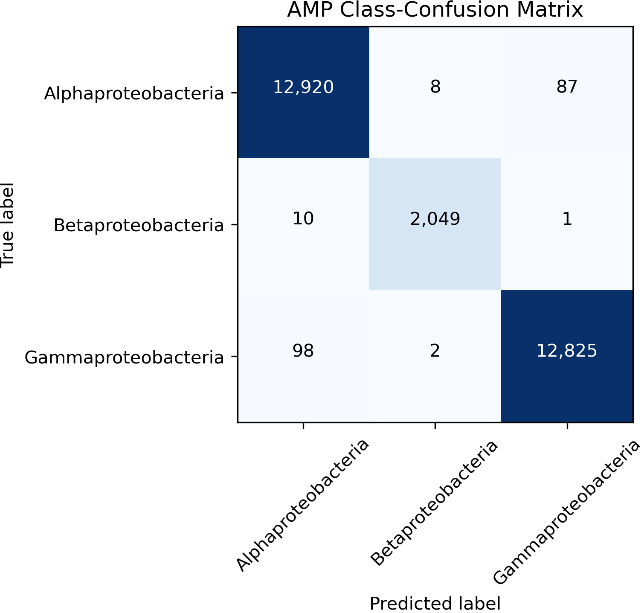


**S11 Fig. Confusion matrix of AMP class data set**


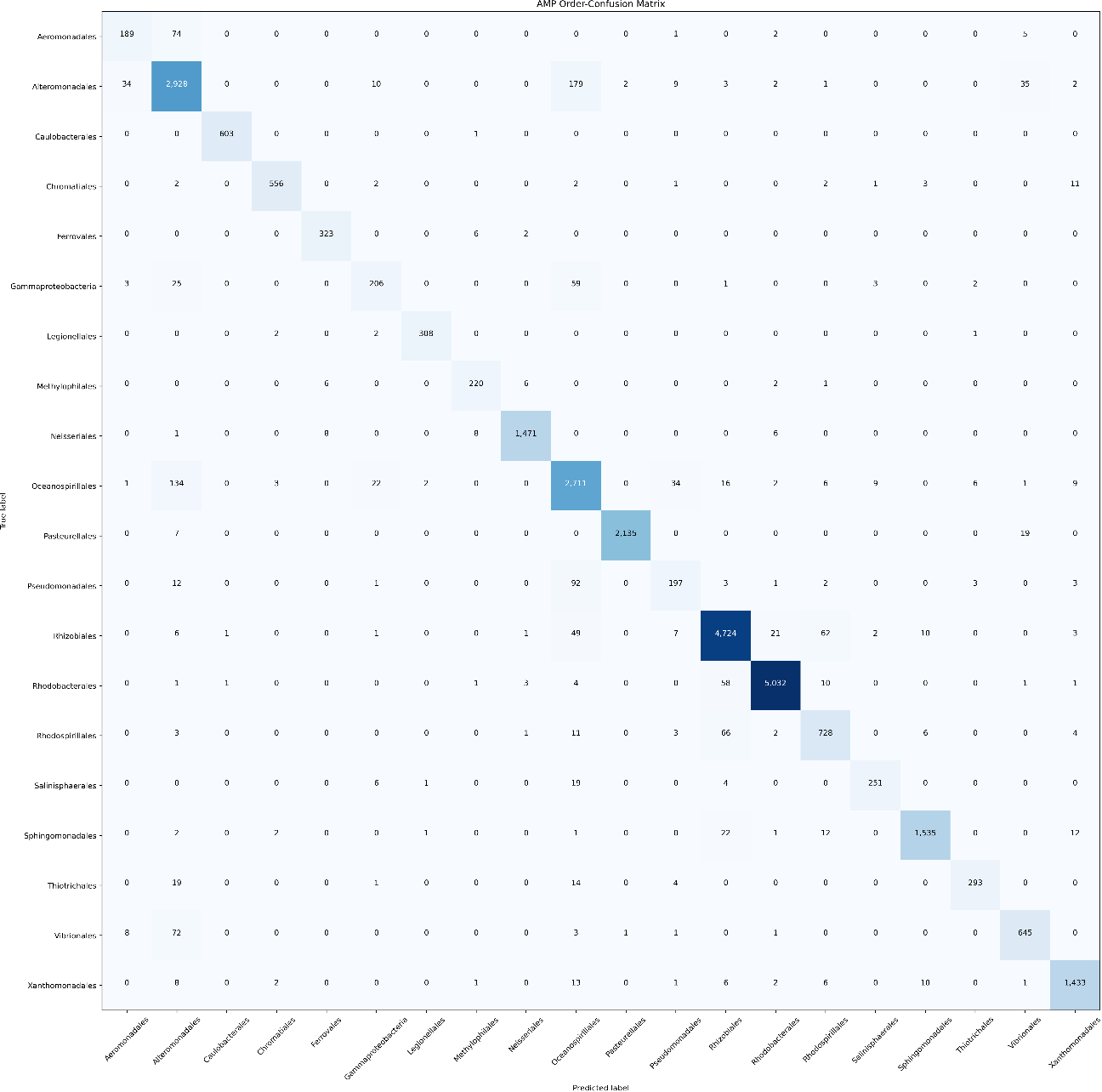


**S12 Fig. Confusion matrix of AMP order data set**


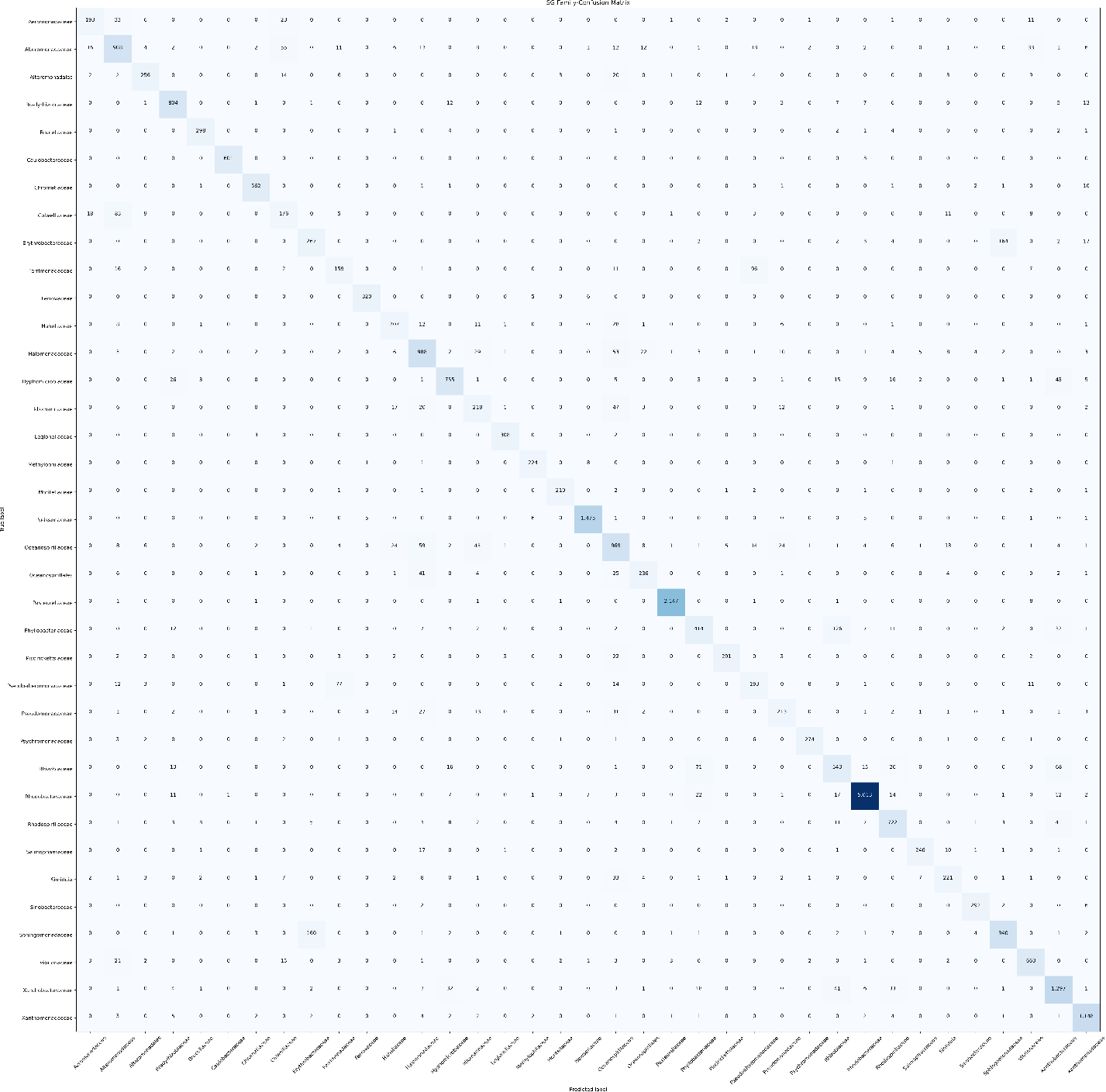


**S13 Fig. Confusion matrix of AMP family data set**

# References

[1] A. Fabijanska and S. Grabowski, “Viral Genome Deep Classifier,” *IEEE Access*, vol. 7, pp. 81297–81307, 2019.

[2] C. M. Nugent and S. J. Adamowicz, “Alignment-free classification of COI DNA barcode data with the Python package Alfie,” *Metabarcoding and Metagenomics*, vol. 4, Sep. 2020.

[3] A. Fiannaca *et al.*, “Deep learning models for bacteria taxonomic classification of metagenomic data,” *BMC Bioinformatics*, vol. 19, no. S7, p. 198, Jul. 2018.

[4] S. Colburn, Y. Chu, E. Shilzerman, and A. Majumdar, “Optical frontend for a convolutional neural network,” *Appl. Opt.*, vol. 58, no. 12, p. 3179, Apr. 2019.

[5] S. Akbari Rokn Abadi, N. Hashemi Dijujin, and S. Koohi, “Optical pattern generator for efficient bio-data encoding in a photonic sequence comparison architecture,” *PLoS One*, vol. 16, no. 1, p. e0245095, Jan. 2021.

[6] A. Vander Lugt, “Signal Detection By Complex Spatial Filtering,” *IEEE Trans. Inf. Theory*, vol. 10, no. 2, pp. 139–45, 1964.

[7] M. L. Yee and D. C. Craft, “Fast DNA sequence alignment using optical computing,” no. November, 1996.

[8] X. Sui, Q. Wu, J. Liu, Q. Chen, and G. Gu, “A Review of Optical Neural Networks,” *IEEE Access*, vol. 8, pp. 70773–70783, 2020.

[9] X. Guo, T. D. Barrett, Z. M. Wang, and A. I. Lvovsky, “Backpropagation through nonlinear units for the all-optical training of neural networks,” *Photonics Res.*, vol. 9, no. 3, p. B71, Mar. 2021.

[10] J. Spall, X. Guo, T. D. Barrett, and A. I. Lvovsky, “Fully reconfigurable coherent optical vector–matrix multiplication,” *Opt. Lett.*, vol. 45, no. 20, p. 5752, Oct. 2020.

[11] R. Ayachi, M. Afif, Y. Said, and M. Atri, “Strided Convolution Instead of Max Pooling for Memory Efficiency of Convolutional Neural Networks,” 2020, pp. 234–243.

[12] “HS7: Fastec Imaging’s HS Series high-speed camera,” *4 october 2021*. [Online]. Available: https://hsi.ca/product/hs7/.
